# Nuclear FAM289-Galectin-1 interaction controls FAM289-mediated tumor promotion in malignant glioma

**DOI:** 10.1186/s13046-019-1393-7

**Published:** 2019-09-06

**Authors:** Xing Rong Guo, Mu Yu Wu, Long Jun Dai, Yu Huang, Meng Ye Shan, Shi Nan Ma, Jue Wang, Hao Peng, Yan Ding, Qiu Fang Zhang, Jun Ming Tang, Xu Zhi Ruan, Dong Sheng Li

**Affiliations:** 1 Hubei Key Laboratory of Embryonic Stem Cell Research, Taihe Hospital, Hubei University of Medicine, Shiyan, 442000 Hubei China; 2 College of Basic Medicine, Hubei University of Medicine, Shiyan, 442000 Hubei China; 3 Department of Integrated Medicine, Affiliated Dong feng Hospital, Hubei University of Medicine, Shiyan, China; 4 Department of Neurosurgery, Taihe Hospital, Hubei University of Medicine, Shiyan, China; 5 Department of Surgery, University of British Columbia, Vancouver, Canada

**Keywords:** Glioblastoma multiforme cell, FAM289, ERK pathway, DNMTs

## Abstract

**Background:**

FAM92A1–289(abbreviated FAM289) is recognized as one of the newly-discovered putative oncogenes. However, its role and molecular mechanisms in promoting cancer progression has not yet been elucidated. This study was performed to reveal its oncogenic functions and molecular mechanisms in human glioblastoma multiforme (GBM) cell models with knockdown or overexpression of FAM289 in vitro and in vivo.

**Methods:**

To elucidate the molecular mechanisms underlying FAM289-mediated tumor progression, the protein-protein interaction between FAM289 and Galectin-1 was verified by co-immunoprecipitation, followed by an analysis of the expression and activity of Galectin-1-associated signaling molecules. Knockdown and overexpression of FAM289 in glioma cells were applied for investigating the effects of FAM289 on cell growth, migration and invasion. The determination of FAM289 expression was performed in specimens from various stages of human gliomas.

**Results:**

FAM289-galectin-1 interaction and concomitant activation of the extracellular signal-regulated kinase (ERK) pathway participated in FAM289-mediated tumor-promoting function. Since the expression of DNA methyl transferase 1 (DNMT1) and DNA methyl transferase 3B (DNMT3B) was regulated by FAM289 in U251 and U87-MG glioma cells, Galectin-1 interaction with FAM289 may promote FAM289 protein into the cell nucleus and activate the ERK pathway, thereby upregulating DNMTs expression. Drug resistance tests indicated that FAM289-mediated TMZ resistance was through stem-like property acquisition by activating the ERK pathway. The correlation between FAM289, Galectin-1 expression and the clinical stage of gliomas was also verified in tissue samples from glioblastoma patients.

**Conclusions:**

Our results suggest that high expression of FAM289 in GBM tissues correlated with poor prognosis. FAM289 contributes to tumor progression in malignant glioma by interacting with Galectin-1 thereby promoting FAM289 protein translocation into the cell nucleus. FAM289 in the nucleus activated the ERK pathway, up regulated DNMTs expression and induced stem-like property gene expression which affects drug resistance of glioma cells to TMZ. This study provided functional evidence for FAM289 to be developed as a therapeutic target for cancer treatment.

**Electronic supplementary material:**

The online version of this article (10.1186/s13046-019-1393-7) contains supplementary material, which is available to authorized users.

## Background

Glioblastoma multiforme (GBM) is the most common and deadly malignant primary tumor of the brain [1]. Although surgical techniques and adjuvant therapies have undergone progressive development for decades, the therapeutic outcomes for treating GBM remain poor [2]. GBM remains an essentially incurable disease, resulting in a death rate of greater than 95% within 5 years of diagnosis. There are several cellular aspects that make this entity a highly lethal and difficult to treat disease. First and foremost, glioblastoma cells are characterized by a nearly unlimited proliferation capacity, marked resistance to chemotherapeutic agents, as well as fatal dissemination into healthy brain tissue [3–5], which are caused by self-renewing properties of cancer stem-like cells (CSCs) [6, 7]. Because of disappointing results with conventional therapies, effective approaches are urgently needed to significantly improve the outcomes for patients with GBM. Considering cancer stem-like cell (CSLC) controlled self-renewal as tumor’s ability to survive the therapeutic treatment and reconstitute tumor cellular heterogeneity, it is critical to identify the molecular mechanisms that regulate this activity and discover more efficient therapeutic treatments.

FAM92A1 gene was first identified by Strausberg et al. in 2002 [8], which is a highly conserved gene located at 8q22.1 that has 10 transcriptional variants and encodes a group of small-molecule nucleoproteins [9, 10]. FAM92A1–289 (FAM289) is the largest transcriptional variant of the FAM92A1 family discovered by our group in 2007 [9]. FAM289 encodes a protein with a Bin-Amphiphysin-Rvs (BAR) domain [11], which is known to bind to lipid membrane surfaces and generate membrane curvature [12–14]. BAR-containing proteins have been shown to play diverse roles in inflammation, cell migration, cell growth and survival [15, 16].

Our previous studies demonstrated that FAM289 retains many oncogenic properties as evidenced by facilitating cell migration, boosting cell proliferation and promoting colony formation in vitro and tumor growth in vivo in cervical carcinoma cells [17] and renal tumor cells [18]. Marcucciet al. found increased FAM289 levels in biopsies of patients with acute myeloid leukemia, with the highest correlation level for poor outcomes [19]. Though the current findings revealed that FAM289 may be a novel gene related to tumorigenesis, the function of FAM289 remains largely uncharacterized, especially in GBM. In this study, we investigated FAM289’s functional significance, molecular mechanisms and clinical implications in GBM, so as to provide cellular and molecular basis for exploring effective molecular targeting therapy for GBM.

## Materials and methods

### Cells and animals

The human glioblastoma multiforme (GBM) U251, DBTRG, U87-MG cell lines were purchased from the American Type Culture Collection (ATCC, Manassas, VA, USA) to be used as target cells in the present study. The human GBM cell lines were maintained as suggested by the ATCC. NCG mice (New generation of NSG-like immune deficient mice) and BALB/c-nude mice (female, 6–8 weeks of age) were purchased from the Model Animal Research Center at Nanjing University (Nanjing, China) and housed in accordance with the National Institutes of Health Guide for the Care and Use of Laboratory Animals. The experimental protocols of the present study were approved by the Animal Care Committee at Hubei University of Medicine (Shiyan, China).

### Patients and clinical specimens

A total of 6 normal brain tissues, 30 early-stage (I–II) and 61 advanced-stage (III–IV) GBM tissue samples were collected from Department of Neurosurgery, Taihe Hospital, Hubei University of Medicine (Shiyan, China) with written informed consent. The ethical approval was granted from Committees for Ethical Review in China Taihe Hospital (Shiyan, China). Pathological diagnosis was made according to the histology of tumor specimens or biopsy and examined by experienced pathologists. Brain cancer tissues and normal brain tissues were stored in liquid nitrogen. The study is compliant with all relevant ethical regulations for human research participants, and all participants provided written informed consent.

### Over-expression of FAM289 in U251 cells

U251 cells were cultured until 70–80% confluency and then transfected with the CMV-SP6-TALEN-GFP-FAM289 vector [17] using the Lipofectamine™ 3000 transfection reagent (Invitrogen, USA) as per the manufacturer’s instructions. After 24 h of incubation, the transfection medium was replaced with fresh medium containing 3.5 μg/ml puromycin to initiate the positive U251 cell screening procedure. The same culture medium was replaced every third day for14 days. On day 14, the U251 cells transfected with CMV-SP6-TALEN-GFP-FAM289 (U251-FAM289^OE^) showed good growth and were observed under a microscope to confirm the presence of green fluorescence. FAM289-over-expressed U251 cells, identified by cloning culture, PCR and western blot assays, were selected for further experiments.

### Lenti-CRISPRv2/Cas9-mediated downregulation of FAM289 in U87-MG cells

The sgRNA-coding cDNAs for targeting the FAM289 gene were designed and synthesized to make the FAM289-sgRNA-Cas9 constructs as described by Wang et al. [20]. The primers including the 20 bp target sequence and *Bsm*BI sticky end were annealed and inserted into the lenti-CRISPRv2 plasmid (Genloci Biotechnologies, China) and digested with *Bsm*BI (NEB, USA). The primer sequences are provided in Additional file 6: Table S1.

FAM289 lenti-CRISPRv2/Cas9 plasmid was used for gene knockdown experiments. To package lentivirus, each lenti-CRISPRv2 plasmid with other components (psPAX2,pMD2.G) were transfected into HEK293T cells using Lipofectamine™ 3000 (Invitrogen, USA). Transfected cells were cultured in DMEM containing 5% FBS, 100 U/ml penicillin and 100 μg/ml streptomycin. Culture media were replaced after 24 h with fresh growth media. Forty-eight hours later, lentiviral particles were concentrated from culture media filtrated with 0.45 μm filters by using the Lenti-X Concentrator (Clontech, USA). Aliquots were stored at − 80 °C until use. To transduce U87-MG cells, 1.0 × 10^5^ cells were plated in each well of a six-well plate, infected with the lentivirus, treated with polybrene for 24 hrsand selected by adding 3.5 μg/ml puromycin to the growth medium for 4–6 days. Lentivirus with a scrambled sequence was used as control.

### RNA interference

FAM289 and Galectin-1siRNA oligonucleotides were transfected using Lipofectamine 3000 (Invitrogen, USA) according to the manufacturer’s instructions. The siRNA target sequences used in this study are shown in Additional file 6: Table S2. All siRNAs were synthesized by Ribobio (RiboBio, China) and were transfected at a final concentration of 10 nM.

### Quantitative real-time PCR

Total RNA was extracted using the TRIzol reagent (Ambion, Austin TX, USA) according to the manufacturer’s recommendations and was quantified by UV spectroscopy. To prepare the RNA for PCR analysis, 2 μg of total RNA was converted to cDNA using the FastQuant RT kit with gDNase (Tiangen Biotech, China). Quantitative real-time PCR was then conducted with a SYBR-Green master mix (Takara Bio, China). The PCR reaction was preceded as follows: 95 °C for 30 s, followed by 40 cycles of 95 °C for 30 s, 60 °C for 30 s and 72 °C for 30 s. The gene expressions were normalized with β-actin. The primers are provided in Additional file 6: Table S3.

### Antibodies and western blot analysis

Cells of each group were collected and lysed by lysis buffer (Beyotime Biotechnology, China). The proteins were extracted and separated by SDS-PAGE, and were then transferred onto PVDF membranes. After blocking with 1% BSA for 1 h at room temperature (RT), the membranes were incubated with antibodies against GFP, Flag FAM289 (FAM92A1 Antibody), anti-Galectin-1, H3, ERK, NF-κB, DNMT1, DNMT3B, Galectin-1, SOX2, OCT4, CD133, C-Myc, GAPDH, anti-pERK1/2 and pNF-κB (The details of antibodies are provided in Additional file 6: Table S4) at 4 °C overnight. After washing 3 times with TBST, the membranes were incubated with the horseradish peroxidase (HRP)-conjugated secondary antibody (1:1000, Beyotime Biotechnology, China) at RT for 1 h. Then the membranes were washed 3 times with TBST and imaged with a gel imaging system (BIO-RAD, USA).

### Co-immunoprecipitation assay

For the co-immunoprecipitation assay, HEK293T cells or U251 cells at a confluency of 60–70% in 6-well plates were transfected with a total of 5 mg plasmids. Twenty-four hours after transfection, cells were washed once in PBS and lysed in buffer A containing 25 mM Tris-HCl, pH 7.6, 150 mM NaCl, 10% glycerol and 1% Triton X-100, supplemented with a protease inhibitor mixture (Roche Molecular Biochemicals, Stockholm, Sweden). Pre-cleared lysates were subjected to anti-Flag M2 immunoprecipitation (Sigma, USA) or protein G magnetic beads (Beyotime Biotechnology, China) following the manufacturer’s instructions. The beads were washed four times with a lysis buffer and the immunoprecipitates were eluted by 2 × SDS sample buffer followed by standard immunoblotting analysis. All the immunoprecipitation assays were performed more than three times and representative results were presented.

### Immunofluorescence

Cultured cells were fixed with 4% paraformaldehyde (Beyotime, China) for 10 min and washed three times with PBS. The cells were permeabilized with 0.1% Trion-X 100 (Beyotime, China) for 20 min, and blocked with 2% bovine serum albumin at RT for 60 min. The cells were incubated with the following antibodies for 60 min at 37 °C: rabbit anti-human FAM289 1:200 (Proteintech, USA), rabbit anti-human Galectin-1 1:200 (Proteintech, USA). After rinsing with PBS three times, cells were incubated with the following secondary antibodies for 60 min: FITC-conjugated mouse anti-rabbit IgG 1:200, or Cy3-conjugated mouse anti-rabbit IgG 1:200 (Beyotime, China). Cell nuclei were counterstained with DAPI (Beyotime, China). Cells were examined under a Confocal Microscope (Olympus, Japan).

### Scratch assay

The scratch assay was used to observe the cell migration. The U251or U87-MG cells were cultured to completely confluent in 6-well plates and a scratch was made across the cell monolayer of each well with a 10 μl pipette tip. The cell monolayer was then washed 3 times with D-PBS and incubated in serum free DMEM at 37 °C with 5% CO_2_ for 48 h. The widths of the scratch were measured and the percentages of the narrow down were compared at 0 h, 24 h and 48 h respectively. The experiment was performed in triplicates.

### Cell migration assay

Cell migration assays were carried out with transwell filters. U251 or U87-MG cells were digested and re-suspended with DMEM medium to 1 × 10^5^cells/ml. Two hundred microliters cell suspension were seeded into an upper chamber of polycarbonate membrane filter inserts with 8-μm pores (Corning, USA). The lower chamber was filled with 500 μl of DMEM medium with 10% FBS. The cells (not migrated) in the upper chamber surface were removed with cotton swabs after incubated at 37 °C for 24 h, and the cells (migrated) on the bottom side of the membrane were fixed with 95% ethanol for 30 min, stained with the 0.1% crystal violet and counted under a microscope. The experiment was performed in triplicates.

### Cell viability assay

Cell viability was also detected by real-time assessment using the xCELLigence real time cell analyzer (RTCA, Roche, Sweden) as previously described [21]. U251 or U87-MG cells were trypsinized and counted using the trypan blue exclusion method along with a haemacytometer and were then re-suspended in culture medium. Background measurements were taken from the wells by adding 100 μl of the same medium to the E-Plate 16. A volume of 100 μl of cell suspension (3 × 10^3^ cells) was then added to the wells to make a final volume of 200 μl. All cells were allowed to settle at the bottom of the wells at RT for 15 min, and were then incubated at 37 °C and 5% CO_2_. The impedance signals were recorded every 15 min for 72hs.

### Xenograft assay

Subcutaneous xenograft model: The U251 and U251-FAM289OE cells were subcutaneously injected into NCG mice (Because of the low degree malignancy, U251 cells could not form tumors in BALB/c-nude or SCID mice, so we used the new generation of NSG-like immune deficient mice for this experiment, *n* ≥ 5 mice each group) separately. The U87-MG and U87-MG-FAM289KD cells were subcutaneously injected into BALB/c-nude mice (5 mice each group) separately. These mice were bred and the sizes of the neoplasms from implanted cells were checked weekly.

Orthotopic model: The U251-Luc cells with/without FAM289 overexpression were injected into NCG mice brains (*n* ≥ 3 mice each group) separately. The sizes of grafted tumors were measured every 7 days using a small animal image system (IVIS, Caliper Life Sciences, Hopkinton, MA, USA) until the mice died. Each test was conducted 15 min after the anesthetized mouse received a 200 μl D-luciferin solution (0.15 mg/ml, Caliper Life Sciences) intraperitoneal injection. The growth rate and size of tumors were analyzed using the radiance value which is proportional to the number of bioluminescence-producing cells.

### Plasmid construction

=To transduce the NLS-FAM289 (possessing the nuclear localization signal (NLS) sequence, 197PHPPKRLRSDPDAC210, from human Gemin-4 (GenBank ID: AAF35283.1), on the C-terminus of GAL-1), NES-FAM289 (possessing the nuclear export signal (NES) sequence, 4INQMFSVQLSL14, from human Staufen-2 (GenBank ID: AAH08370.1), on the C terminus of FAM289) [22], each cDNA was produced by PCR, confirmed by sequencing and ligated into pEGFP-N1 vector, which was generated in our laboratory. The primers are provided in Additional file 6: Table S5.

### Tumorsphere formation assay

The U251 and U87-MG cells in good growth condition were counted and 1 × 10^3^ cells were plated into 12-well Ultra Low Cluster plates (Corning). The cells were cultured with a serum-free DMEM/F12 medium containing 100 U/ml penicillin, 100 μg/ml streptomycin, 20 ng/ml epidermal growth factor (EGF), 10 ng/ml basic fibroblast growth factor (bFGF) (Gibco, USA) at 37 °C for 10–14 days. Three replicates were set for each type of cell. The culture medium was replaced according to the cell growth rate and the color changes of the culture medium. The cultures were photographed regularly under the microscope to observe tumor-sphere formation.

### Flow cytometry analysis

To determine the cell surface marker expression, cells were harvested with trypsin for flow cytometry analysis. After being blocked with 2.5% FBS, a total of 5 × 10^5^ cells were re-suspended in 200 μl PBS for each reaction. Cells were incubated on ice for 30 min with the following anti-human antibodies, which were conjugated with fluorescein phycoerythrin (PE): CD133-PE, Ki67-PE and CXCR4-PE (BioLegend, USA). Rabbit IgG1-PE (Beyotime, China) was used as the isotype control. Cells were analyzed by flow cytometry (Beckman Coulter, USA).

To determine the intracellular marker expression, cells were harvested and then fixed in a 0.5 ml/tube Fixation Buffer in the dark for 20 min at RT, followed by a 1x wash with Cell Staining Buffer. Cells were then re-suspended in Cell Staining Buffer and stored in 90% FCS/10% DMSO at − 80 °C. The fixed/permeabilized cells were re-suspended in residual Intracellular Staining Perm Wash Buffer and were added a predetermined optimum concentration of PE-conjugated antibody of Ki67 (BioLegend, USA) or rat IgG2b-PE isotype (BioLegend, USA) for 20 min in the dark at RT. Cells were analyzed by flow cytometry (Beckman Coulter, USA).

### Apoptosis and cell cycle assays

Cells in early and late apoptotic stages were quantified using an Annexin V-APC/PE double staining assay (Beyotime, China). Cells were collected and re-suspended in 500 μl binding buffer at 1 × 10^6^ cells/ml, followed by staining with 5 μl Annexin V and 5 μl PE in the dark at RT for 15 min. Stained cells were immediately examined using a FACS flow cytometry analyzer (Beckman Coulter, USA) with wavelength emission filters of 488–530 nm for green fluorescence of Annexin V (FL1) and of 488–630 nm for red fluorescence of PI (FL2). For the cell cycle assay, 3 × 10^5^ cells were seeded into a 6-well plate. After 24 h incubation, the cells were collected and fixed with 75% cold ethanol (1 ml PBS and 3 mL absolute ethanol) at − 20 °C overnight. After that, the cells were incubated with 500 μl propidium iodide (PI, 100 μg/ml) for 30 min at RT in the dark and analyzed using the FACScan flow cytometer (Becton Dickinson, Franklin Lakes, NJ, USA). The data were analyzed with ModFitLT V2.0 software (Becton Dickinson, USA). All experiments were performed three independent times.

### Immunohistochemical staining

Briefly, 5-μm serial sections were dewaxed in xylene and rehydrated through graded alcohols. Endogenous peroxidases were blocked with 3% H_2_O_2_ for 30 min, and antigens were retrieved through microwaving slides. After cooling and washing, slides were blocked with goat serum (1:10; Zymed antibody diluent; 30 min). The sections were incubated with primary antibodies anti-FAM289 (FAM92A1 Antibody, 1:200, Atlas, Bromma, Sweden) and anti-Galectin-1(1:200, Proteintech, USA) at 4 °C overnight and then were incubated with HRP-conjugated secondary antibodies followed by the Liquid DAB Substrate Chromogen System according to the manufacturer’s instructions. The sections were examined under a fluorescence microscope (Olympus, Japan).

### Statistical analysis

In general, unpaired two-tailed Student t test and one-way ANOVA were used to make inter-group comparisons. The Kaplan-Meier method was used to estimate overall survival. All statistical analyses were performed with SPSS (version 16.0) and GraphPad (Version 5.0). All results were presented as mean ± SD (standard deviation) with a *P* value < 0.05 considered statistically significant.

## Results

### FAM289 is highly expressed in GBM cells and tissues

**Fig. 1:**
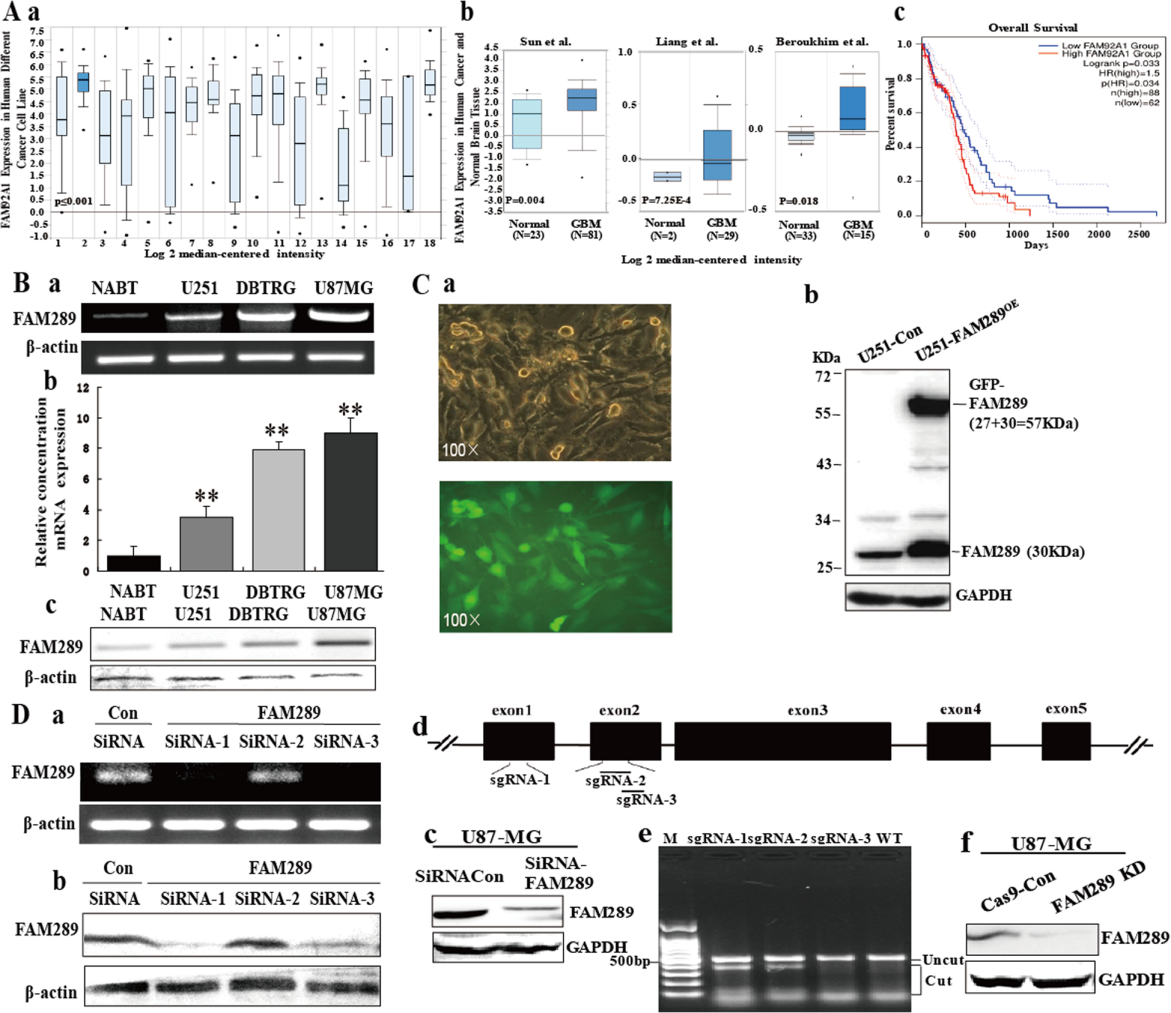
FAM289 was overexpressed in GBM cell lines and tissues. (A) FAM92A1 expression levels were increased in human brain cancer cells and tissues by analyzing the GBM RNA sequencing dataset from TCGA. (a) FAM92A1 expression levels were increased in human brain cancer cells compared to other kinds cancer cell lines (1. Bladder Cancer (*n* = 21) 2. Brain and CNS Cancer (*n* = 64) 3. Breast Cancer (*n* = 56) 4. Colorectal Cancer (*n* = 56) 5. Esophageal Cancer (*n* = 25) 6. Gastric Cancer (*n* = 35) 7. Head and Neck Cancer (*n* = 41) 8. Kidney Cancer (*n* = 21) 9. Leukemia (*n* = 83) 10. Liver Cancer (*n* = 29) 11. Lung Cancer (*n* = 166) 12. Lymphoma (*n* = 61) 13. Melanoma (*n* = 57) 14. Myeloma (*n* = 26) 15. Ovarian Cancer (*n* = 44) 16. Pancreatic Cancer (*n* = 44) 17. Prostate Cancer (*n* = 7) 18. Sarcoma (*n* = 39)). (b) Geometric mean of the FAM92A1 expression was significantly higher for GBM tissues compared with normal brain samples in three independent datasets. (c) Kaplan–Meier analysis of overall survival curves of FAM2A1 expression and clinical outcomes from the TCGA database. (B) The mRNA levels of FAM289 in different degrees of malignancy GBM cells lines (U251, DBTRG, U87-MG) was analyzed by western blotting (a) and qRT-PCR (b). (C) Overexpression of FAM289-GFP fusion protein in U251 cells was verified by fluorescence microscopy (a) and western blotting (b). (D) FAM289 was knocked down in U87-MG cell lines by siRNA transfection (a-c) or LentiCRISPRv2 –FAM289 infection (d-f). NABT: normal brain tissue; KD: knock down; siRNA: small interfering RNA; sgRNA: small guide RNA; Con: control; OE: overexpression; M: 100 bp DNA ladder

To determine the clinical significance of FAM92A1 for GBM patients, we first analyzed several publicly available RNA datasets of GBM from The Cancer Genome Atlas (TCGA) and Oncomine database (www.oncomine.org). As shown in Fig. 1a, we found that FAM92A1 level was up-regulated in human brain and CNS cancer cells compared to other types cancer cell lines (Fig. 1Aa), but the expression of FAM92A1 in human normal brain was not higher as compared with other normal tissue [23]. The geometric mean of the FAM92A1 expression was significantly higher for GBM tissues compared with normal brain samples in each of the three validation datasets (Fig. 1Ab). Meanwhile, we evaluated the correlation between FAM2A1 expression and clinical outcomes from the TCGA database using the Kaplan Meier Plotter (www.gepia.cancer-pku.cn). As shown in Fig. 1Ac GBM patients with high FAM92A1 expression showed shorter overall survival (*n* = 150, *P* < 0.05). Taken together, these public database results implied that high expression of FAM92A1 may be associated with the progression and metastasis of GBM.

FAM289 (FAM92A1–289) is the largest transcriptional variant of the FAM92A1 family discovered by our group in 2007 [9]. Our previous studies demonstrated that FAM289 retains many oncogenic properties in cervical carcinoma cells [17] and renal tumor cells [18]. So we speculate that FAM289, a transcriptional variant of FAM92A1, contributes to tumorigenesis in malignant gliomas. To confirm our hypothesis, we detected the expression levels of FAM289 in GBM cancer cell lines (U251, DBTRG, U87-MG cells) and normal brain tissue (NABT) by qRT-PCR and western blotting. FAM289 expression was significantly increased in GBM cell lines with high degree of malignancy (U87-MG) compared with low metastatic GBM cell lines (U251) and NABT (Fig. 1B). To elucidate the role of FAM289 in GBM progression, a stable transfection method was used to increase FAM289 expression in the human U251cell line, which has low FAM289 expression at both RNA and protein levels (Fig. 1C). As shown in Fig. 1Bb, a 61-KDa of GFP-FAM289 recombinant protein and a 30-KDa of endogenous FAM289 protein were detected in FAM289 over-expressed U251 cells (U251-FAM289^OE^ cells). Interestingly, compared with the control, we found that the GFP-FAM289 fusion protein could activate endogenous FAM289 expression. Moreover, the U251-FAM289^OE^ cells showed strong green fluorescence and presented good growth condition (Fig. 1C). RNA interference and lentivirus-mediated CRISPR-Cas9 methods were used to reduce FAM289 expression in the U87-MG cell line, which has a high level of FAM289 protein expression. First, we screened the activity of FAM289 siRNA1–3 in HEK239T cells, and found that siRNA1 and siRNA3 had better inhibitory effect. Then we used siRNA1 and siRNA3 combinations to interfere with FAM289 expression and also found their significant inhibition in U87-MG cells (Fig. 1Da-c). At the same time, three sgRNAs were designed pointed at exon 1 and exon 2 of FAM289 (Fig. 1Dd). After infecting cells with corresponding lentivirus mediated CRISPR-Cas9 plasmids, sgRNA1 showed the best activity by T7E1 digestion (Fig. 1De). The effect of sgRNA1-mediated knock down was checked by western blot (Fig. 1Df). Taken together, this data demonstrated that the U251-FAM289^OE^ (FAM289 stably-overexpressed cell line) and U87-MG-FAM289KD (FAM289 stably-knocked down cell line) were successfully established.

### FAM289 is involved in proliferation and migration of GBM cells

**Fig. 2:**
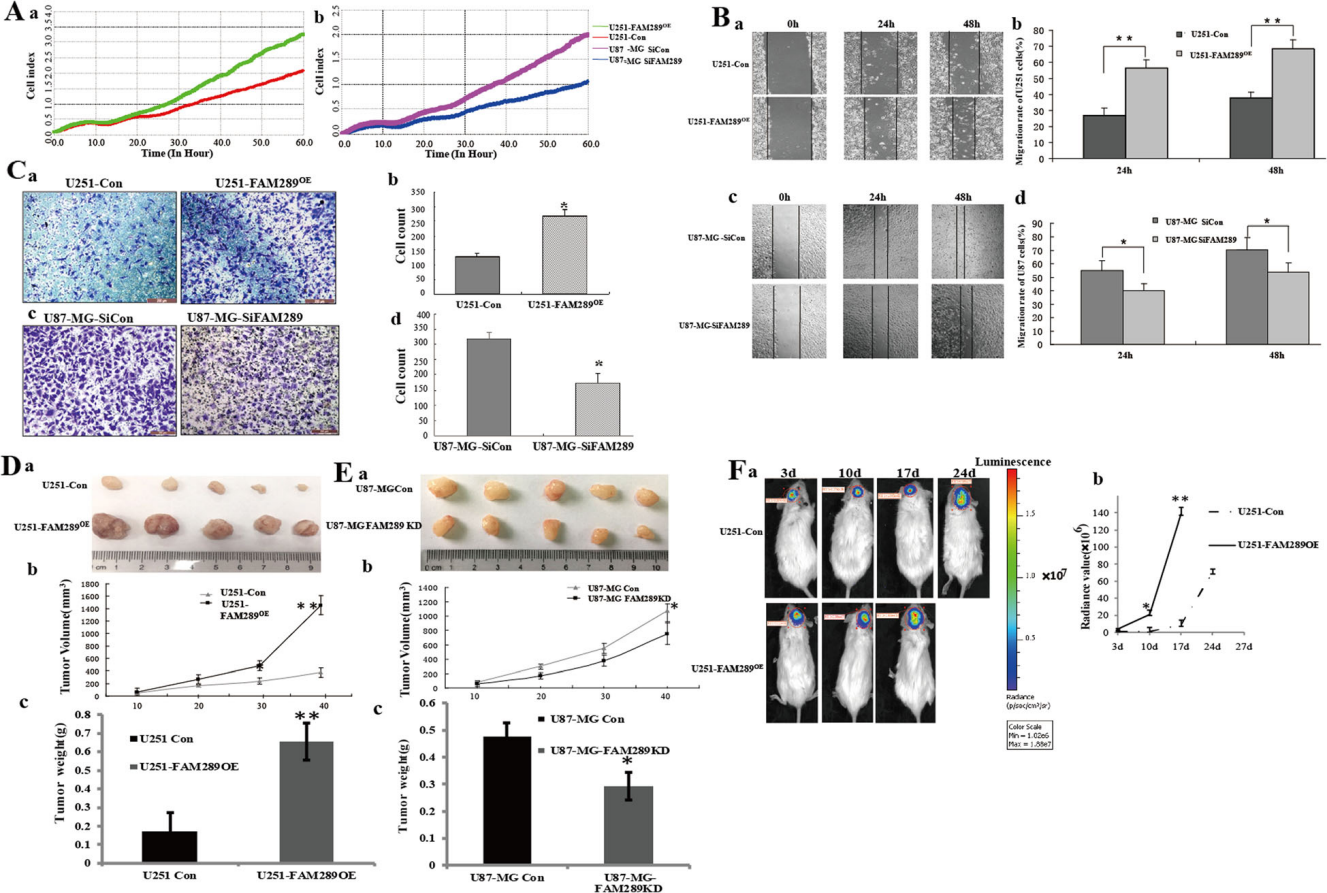
FAM289 is involved in GBM cells proliferation and migration in vitro and in vivo. (A) Real-time monitoring of FAM289-induced proliferation in U251 (a) or U87-MG (b) cells. Cell index was automatically recorded with the xCELLigence real-time cell analyzer (RTCA) every 15 min until the end of the experiment (60 h). Each tracing represents an average of three parallel assessments. (B) The effect of FAM289 on migrating capacity in U251 (a&b) and U87-MG (c&d) cells. The representative pictures were taken at 0 h, 24 h and 48 h after scratching. The differences were significant both at 24 h and at 48 h. (C) Transwell assay of FAM289-induced migrationin U251 (a&b) and U87-MG (c&d) cells. Images were obtained at 48 h. Data are presented as the mean ± SEM of three independent experiments. (D) Ectopic expression of FAM289 accelerated growth of FAM289 xenografts in NCG mice (*n* ≥ 5) as compared to controls. The representative tumor photos and summary volume & weight were presented in (a), (b) and (c) respectively. (E) Knockdown of FAM289 decelerated growth of U87-MG cell-derived xenografts in nude mice (n ≥ 5) as compared to controls (*p < 0.05 and **p < 0.01 vs. control). The representative tumor photos and summary volume & weight were presented in (a), (b) and (c) respectively. (F) The U251-Luc cells with/without overexpression FAM289 (2 × 10^6^ each) were subcutaneously injected into the brain of NCG mice (*n* ≥ 3) and the sizes of neoplasms formed were measured with an in vivo imaging system until mice died (usually 3~4 weeks). (a) Serial pictures taken at different time points; (b) The changes of luminescence radiance values of the neoplasms formed from the injected U251-Luc cells with/without overexpression FAM289. The difference was significant (*p < 0.05 and **p < 0.01 vs. control)

FAM289 upregulation significantly increased the proliferation, migration, and invasion abilities of U251 cells compared with the control. Meanwhile, knockdown FAM289 expression by siRNA apparently reduced the proliferation, migration, and invasion of U87-MG cells (Fig. 2A-C) (P<0.05). In order to directly assess the effect of FAM289 on tumor growth, U251 Con and U251-FAM289^OE^ cells were subcutaneously implanted into NCG mice, while U87-MG Con and U87-MG-FAM289KD cells were subcutaneously implanted into BALB/c-nude mice separately in the present study. As shown in Fig. 2D, the mean tumor size and weight of the neoplasms formed with U251-FAM289^OE^ cells was significantly bigger (*P* < 0.05), while markedly reduced in the FAM289-KD U87-MG-derived xenografts compared with that of the controls (Fig. 2E).

The orthotopic model was used to study the effect of FAM289 on the growth of glioma. LUC (Luciferase)-labeled U251 Con and U251-FAM289^OE^ cells were injected into the brain of female NCG mice. Tumor development was monitored by IVIS on day 3, 7, 17 and 24 after implantation until mice died. The imaging assay indicated that the size of the neoplasms formed from U251-FAM289^OE^ cells was significantly bigger than those from the wild U251-Con cells (*P* < 0.01) (Fig. 2F & Additional file 1: Figure S1), and the survival time of mice injected U251-FAM289^OE^ cells (22–24 days) was also significantly shorter than that of control group (25–27 days). This data implied that FAM289 plays a pivotal oncogenic role in human glioblastoma multiforme carcinogenesis.

### Identification of Galectin-1 as a FAM289-interacting protein

**Fig. 3:**
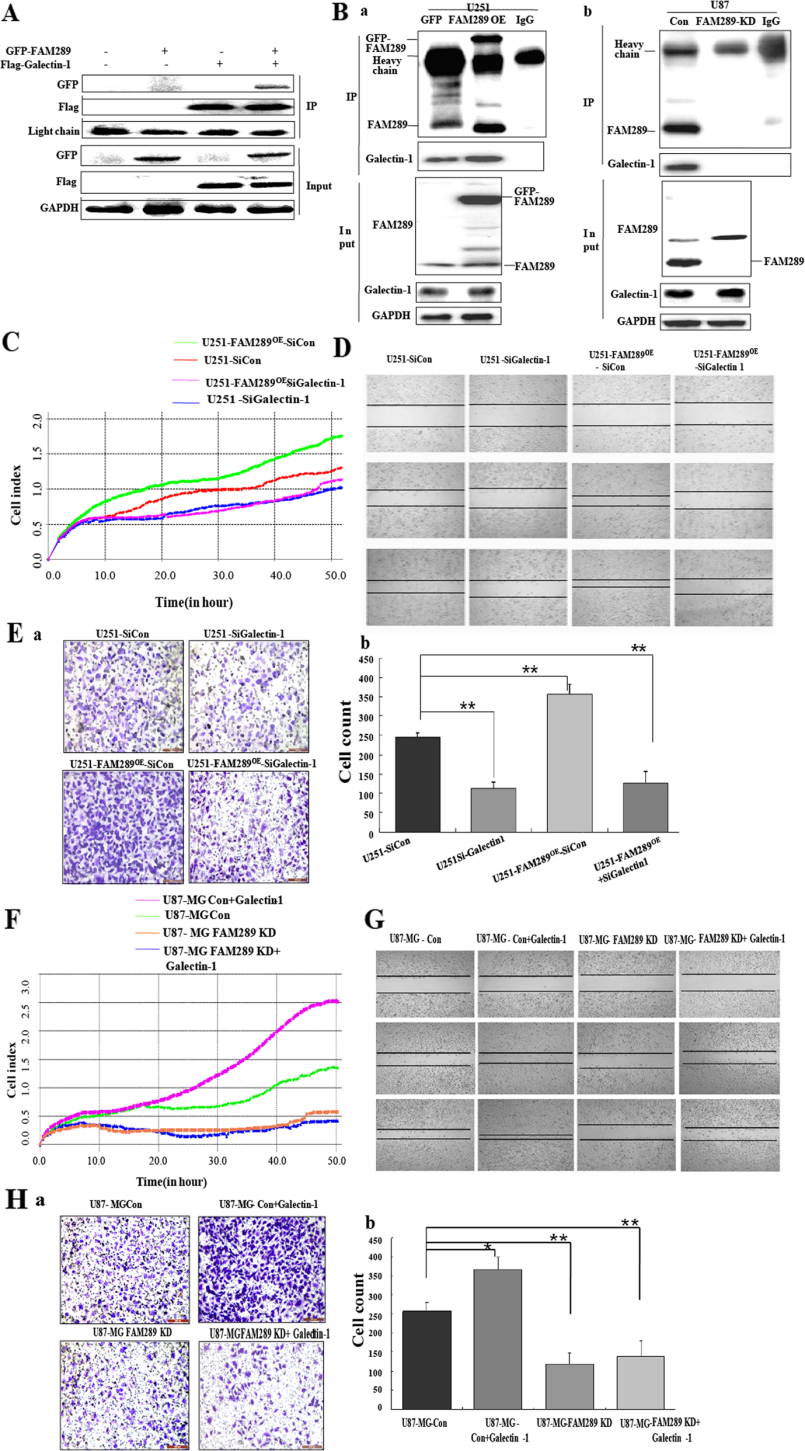
FAM289 promotes proliferation and migration of GBM cells dependent on the interaction with Galectin-1 protein. (A) Exogenous FAM289 interacts with Galectin-1. HEK293T cells were transfected with peGFP-FAM289 and Flag-tagged Galectin-1 plasmids. Co-IP experiment was performed using anti-Flag antibody. (B) Endogenous FAM289 interacts with plasmid-produced Galetin-1. Co-IP experiment was performed using anti-FAM289 antibody in U251 and U87-MG cells. IgG was used as the negative control. The immunoprecipitates were analyzed via western blotting with anti-FAM289 and anti-Galectin-1 antibodies. (C-H) The effect of FAM289/Galectin-1 interaction on the tumor-promoting functions of FAM289.SiRNAs specifically targeting Galectin-1 were transfected into U251 con and U251-FAM289^OE^ cells to knockdown endogenous Galectin-1.These cells’ proliferation and migrating capacity were analyzed by xCELLigence RTCA (C), scratch assay (D) and transwell assay (E). Galectin-1vectors were transfected into U87-MG con and U87-MG-FAM289 KD cells, and these cells’ proliferation and migrating capacity were analyzed by xCELLigence RTCA (F), scratch assay (G) and transwell assay (H). Each tracing represents an average of three parallel assessments. Scale bars. *P < 0.05, **P < 0.01 vs controls

In order to explore the mechanisms underlying FAM289-regulated tumor progression, we previously identified FAM289 interacting partners by screening a yeast two hybrid approach [24]. Galectin-1 was repeatedly recovered by screening a Hela cells cDNA library [24] and classified as a proto-type of galectin, which is well known to be associated with GBM progression via processes of migration [25], invasion [26], angiogenesis [27, 28] and immune escape [29]. To determine whether FAM289 binds galectin-1 in mammalian cells, we initially conducted co-immunoprecipitation experiments using transfected HEK293T cells. Cell lysates from HEK293T cells which express Flag-Galectin-1 and GFP-FAM289 were immunoprecipitated with Flag antibody and analyzed by western blotting. As shown in Fig. 3A, FAM289 specifically interacted with Galectin-1. Then, we performed a co-IP assay to test the interaction between endogenous FAM289 and Galectin-1 (Fig. 3B). A significant amount of Galectin-1 protein was precipitated in U251-FAM289^OE^ cells (Fig. 3Ba), while it markedly reduced in the FAM289-KD U87-MG cells compared with that of the controls (Fig. 3Bb). These results indicated that FAM289 could interact with Gaectin-1 in GMB cells.

### FAM289 and Galectin-1 interdependently regulate tumor proliferation, migration and invasion via ERK and NF-kB signaling pathways

**Fig. 4:**
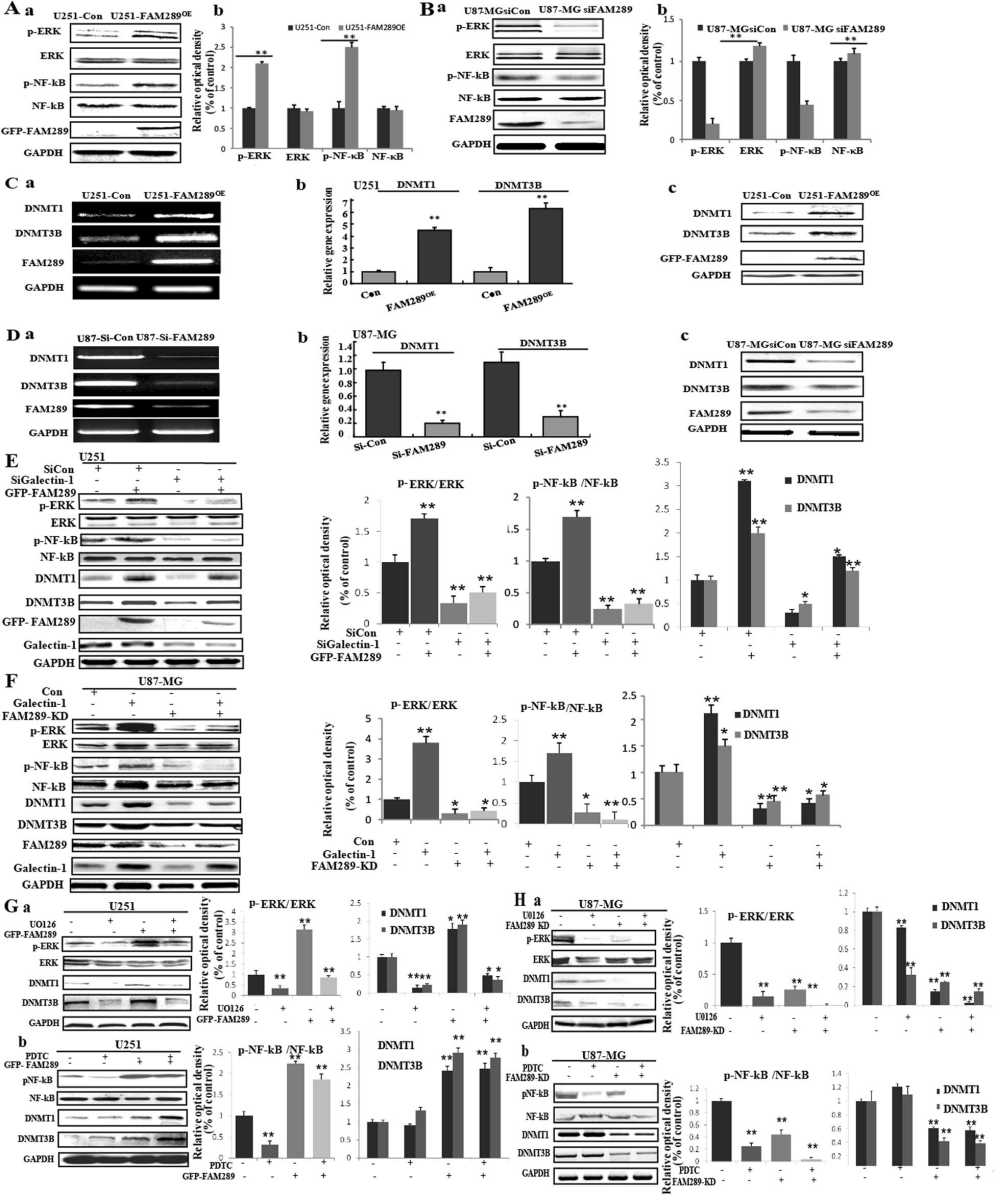
FAM289 and Galectin-1 interdependently regulate tumor proliferation and migration via ERK and NF-kB signaling pathways. (A) FAM289 upregulation increased p-ERK and p-NF-kB expression in U251 cells. (B) FAM289 decreased p-ERK and p-NF-kB expression in U87-MG cells. (C&D) Screening of more than 40 genes downstream of ERK and NF-kB by qPCR and western blotting. The expressions of DNMT1and DNMT3B were regulated by FAM289 in U251 and U87-MG cells. (E) Protein levels of p-ERK, p- NF-kB, DNMT1 and DNMT3B in Galectin-1 siRNA1- and control siRNA-transfected U251 con or U251-FAM289^OE^ cells were detected by western blotting. (F) Protein levels of p-ERK, p- NF-kB, DNMT1and DNMT3B in Galectin-1-flag- and control plasmid-transfected U87-MG con or U87-MG-FAM289KD cells were detected by western blotting. (G&H) The effects of MAPK and NF-kB inhibitors on FAM289-induced expression of DNMT1 and DNMT3B. U251 and U87-MG cells were treated with or without U0126 (U, 1 μM), PDTC (SP, 1 μM) for 24 h and subjected to western blot analysis. *n* = 3, *p < 0.05, * *p < 0.01 vs. Control (−)

To determine whether FAM289 promotes tumorigenesis in malignant glioma by a mechanism dependent on its interaction with galectin-1, we performed RTCA, scratch and cell migration assay experiments to analyze the relationship between FAM289 and Galectin-1 by overexpression or knockdown of their expression in U251 and U87-MG cells. The result of knockdown or overexpression of Galectin-1(Additional file 2: Figure S2) and FAM289 by different combinations indicated that Galectin-1 knockdown could significantly inhibit the proliferation, migration and invasion in U251 cells with FAM289 overexpression (Fig. 3C-E), while overexpression of Galectin-1 did not reverse the inhibitory effect of FAM289 knockdown on cell proliferation, migration and invasion (Fig. 3F-H). Galectin-1 is a critical scaffolding protein and a major regulator of MEK/ERK, NF-kB, PI3K/Akt, SAPK/JNK and other important signaling pathways. Therefore, we next investigated whether FAM289 could modulate these pathways. Our results showed that FAM289 upregulation increased p-ERK and p-NF-kB expression in U251 cells, whereas FAM289 decreased p-ERK and p-NF-kB expression in U87-MG cells (Fig. 4A&B). No significant difference in p-AKT and p-JNK expression was observed with changes in FAM289 expression (data not shown). We first screened more than 40 genes related to tumor proliferation, migration or downstream of ERK and NF-kB by RT-PCR (Primers are listed in Table S1, data are shown in Additional file 3: Figure S3), and then reconfirmed the altered genes by q-RT-PCR and western blot (Fig. 4C&D). We found that the expression of DNA methyl transferase 1 (DNMT1) and DNA methyl transferase 3B (DNMT3B) were regulated by FAM289 in U251 and U87-MG glioma cells (Fig. 4C&D). These observations prompted us to consider whether DNMTs expression is induced by ERK or NF-kB and associated with FAM289-Galectin-1 interaction. To test this point, we knocked down or overexpressed Galectin-1 in U251-FAM289^OE^ or U87-MG-FAM289 KD cells. The expected results were obtained as DNMT1 and DNMT3B expression was increased by p-ERK or p-NF-kB, which was controlled by the interaction of FAM289 with Galectin-1 (Fig. 4E&F). Last, we wondered whether FAM289 regulates DNMT1and DNMT3B expression by activating ERK or NF-kB pathways. To address this point, we treated U251 and U87-MG cells with ERK or NF-kB inhibitors and detected the expression changes of DNMT1 and DNMT3B respectively. After treatment with ERK inhibitor U0126, we found that overexpression of FAM289 could counteract the inhibition of pERK and its downstream DNMTS protein expression in U251 cells, while FAM289 knockdown could further promote the inhibition of pERK and its downstream DNMTs protein expression in U87-MG cells (Fig. 4G). The results of treatment with PDTC, an inhibitor of NF-κB, showed that although FAM289 regulated PDTC’s inhibitory effect on the expression of p-NF-κB was regulated by FAM289 without affecting the expression of DNMTs (Fig. 4H). The results indicated that FAM289 regulated DNMTs expression depended on ERK activation rather than NF-κB activation.

### FAM289 protein localized in nucleus could activate ERK signaling pathway depending on its interaction with Galectin-1

**Fig. 5:**
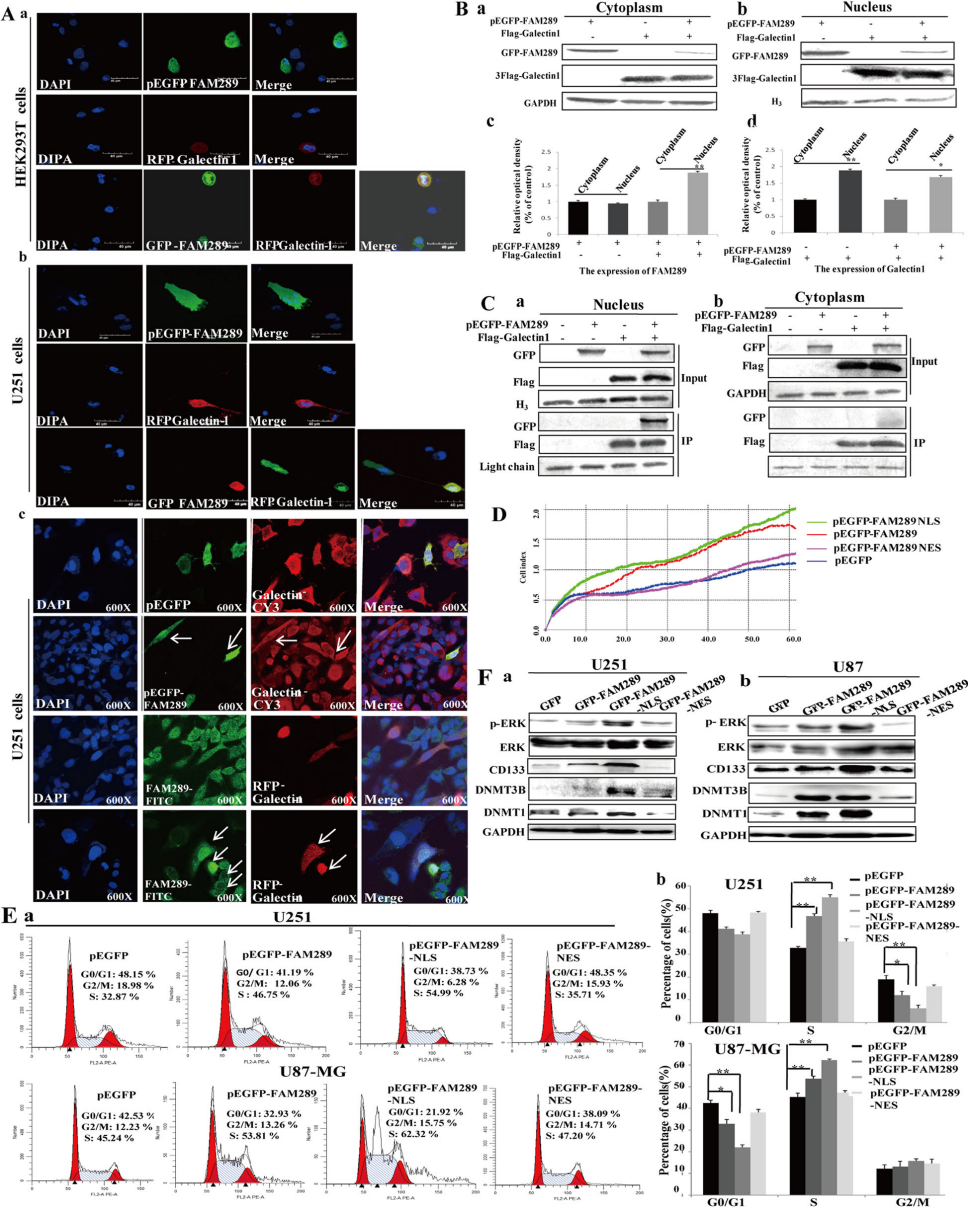
FAM289 protein localized in nucleus could activate ERK signaling pathway depending on its interaction with Galectin-1. (A) Galectin-1 could affect the intracellular localization of FAM289 protein and facilitate its entry into the nucleus. HEK293T and U251 cells were transfected with RFP-galectin-1 and GFP-FAM289 alone or together and stained with DAPI. Scale bars, 40um. (B) Cytoplasmic or nuclear fractions from HEK293T cells transfected with Flag-Galectin-1plasmid or GFP-FAM289 plasmid were immunoblotted with anti-Flag or anti-GFP. Purity of the nuclear and cytoplasmic fractions was verified by immunoblotting using anti-H3 or anti-GAPDH. (C) FAM289 and Galectin-1 mainly interact with each other in the nucleus. Cytoplasmic or nuclear fractions from HEK293T cells transfected with Flag-Galectin-1, GFP-FAM289 or both plasmids were extracted and Co-IP experiment was performed using anti-Flag antibody. (D) pEGFP-FAM289, pEGFP-FAM289-NLS, pEGFP-FAM289-NES and control vectors were transfected into U251 cells. These cells’ proliferation was analyzed by xCELLigence RTCA. (E) The cell cycle was analyzed by flow cytometry after PI staining. The data were processed with ModFit LT program. (F) pEGFP-FAM289, pEGFP-FAM289-NLS, pEGFP-FAM289-NES and control vectors were transfected into U251 and U87-MG cells. The lysates were immunoblotted with anti-ERK, anti-pERK, anti- DNMTS and anti-CD133 antibodies respectively

To gain insight into the molecular mechanisms underlying the pro-tumorigenic action of FAM289 by its interaction with Galectin-1, we first examined the regulation of FAM289 and Galectin-1 on each other’s expression and found that they did not affect each other’s expression (Additional file 4: Figure S4). Galectin-1 has many different functions, dependent on its protein localization in cells. So, we speculate whether FAM289 regulates malignant behavior of tumors by affecting the intracellular localization of Galectin-1. When GFP-FAM289 or RFP-Galectin-1 was overexpressed alone, the two proteins were evenly distributed in the nucleus and cytoplasm. However, when both proteins were overexpressed at the same time in one cell, we were surprised to find that Galectin-1 could affect the intracellular localization of FAM289 protein and facilitate its entry into the nucleus, rather than FAM289 affecting the intracellular localization of Galectin-1protein in HEK293T and U251 cells (Fig. 5Aa&b). Then we overexpressed GFP-FAM289 or RFP-Galectin-1 separately, and found that overexpression of RFP-Galectin-1 could promote the transfer of endogenous FAM289 protein to nucleus, whereas GFP-FAM289 did not affect the distribution of endogenous Galectin-1 protein in U251 cells as detected by immunofluorescence (Fig. 5Ac), Consistent results were obtained by western blot using cytoplasmic or nuclear fractions from U251 cells transfected with pEGFP-FAM289 or Flag-Galectin-1 plasmids (Fig. 5B). Co-IP results showed that FAM289 and Galectin-1 mainly interact with each other in the nucleus (Fig. 5C). Our observations above prompted us to consider whether FAM289 protein’s function might be regulated by altering its distribution between the nucleus and cytoplasm in GBM cells. To test this idea, we overexpressed FAM289 constructs tagged either with a nuclear localization or a nuclear export signal (NLS and NES, respectively; Additional file 5: Figure S5). Nuclear resident FAM289–NLS strongly promotes cell proliferation by reducing the G0/G1 phase and increasing the S phase of cell cycle, while cytoplasmic FAM289–NES did not affect cell cycle and cell proliferation (Fig. 5D&E). On the other hand, ERK phosphorylation and DNMTS expression can only be regulated by FAM289–NLS (Fig. 5F). Collectively, these results suggest that Galectin-1 interaction with FAM289 promotes FAM289 protein into the cell nucleus and activates the ERK pathway, thereby upregulating DNMTs expression, and nuclear FAM289-Galectin-1 interaction controls the tumor-promoting effects of FAM289 in malignant glioma.

### FAM289-mediated TMZ resistance is through stem-like properties acquisition by ERK pathway

**Fig. 6:**
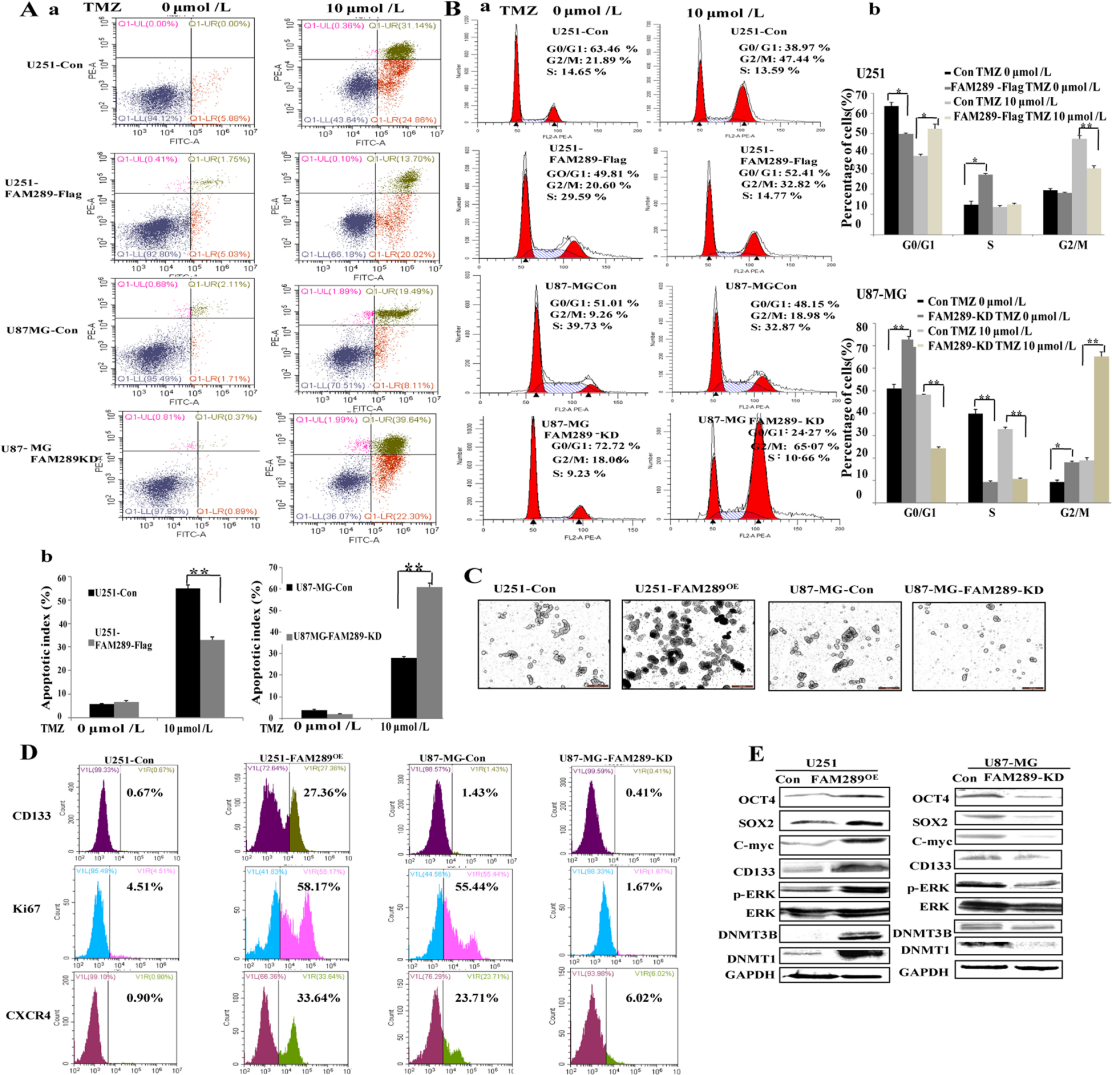
FAM289-mediated TMZ resistance is through stem-like property acquisition via ERK pathway. (A) The apoptosis and cell cycle of U251 and U87-MG cells treaded with TMZ were analyzed by flow cytometry. The cell apoptosis was determined by Annexin V-fluorescein isothiocyanate (FITC)/PI staining. The percentages of Annexin-V-positive cells were indicated. (B) The cell cycle was analyzed by flow cytometry after PI staining. The data were processed with ModFit LT program. (C) The in vitro colony formation in cultured U251 and U87-MG cells. Images were taken on day 10 under an inverted microscope. (D) The cell lysates from FAM289-OE U251 and FAM289-KD U87 cells were immunoblotted with anti-stem-like property factors (OCT4, c-Myc, SOX2, CD133), anti-ERK, anti-pERK and anti-DNMTs antibodies. Corresponding wild type cells were used as controls. (E) The proportion of stemness factor CD133, Ki67 and Chemokine CXCR4 expression was analyzed by flow cytometry

Temozolomide (TMZ), as a first-line therapy for GBM, is frequently limited in durability of treatment response owing to chemoresistance. Therefore, identifying the practical mechanisms of TMZ resistance could provide potential molecular targets for GBM therapy. To determine whether FAM289 is associated with TMZ resistance, GBM cells were transfected with FAM289 plasmid or knocked down by lentivirus. We used flow cytometry to assess the apoptotic rate and cell cycle of FAM289-overexpressed or FAM289-knocked down cells in response to TMZ. Our results demonstrated that ectopic expression of FAM289 led to a significant reduction in apoptosis in U251 cells in the presence of TMZ. Conversely, FAM289 knockdown sensitized GBM cells to TMZ treatment as evidenced by an increased number of apoptotic cells in U87-MG cells (Fig. 6A). PI staining revealed that the overexpression of FAM289 reduced the percentage of cells in the G2/M phase and decreased the percentage of cells in the G0/G1 phase. The ectopic expression of FAM289 could reduce G2/M arrest in TMZ-treated U251cells, showing a significant increase in G2/M-phase arrest compared with FAM289-knocked down cells (Fig. 6B). It is therefore plausible that FAM289 may regulate TMZ resistance in human glioma cells. As accumulating evidence had shown that chemotherapy failure may be caused by the existence of cancer stem cells (CSCs) [30–32], we speculated that FAM289 may mediate TMZ resistance through stem-like properties acquisition. To examine this notion, U251 and U87-MG cells were subjected to tumor-sphere formation assay with serum-free culture for 10 days. Our results showed that the cells with ectopic FAM289 expression significantly enhanced the tumor-sphere forming ability (Fig. 6C) and increased the proportion of stemness factor CD133, Ki67 and chemokine CXCR4 expression (Fig. 6D). Many reports have suggested that the MEK/ERK pathway could regulate stemness factors such as SOX2 and OCT4 [33, 34]. Our results demonstrated that FAM289 could activate the ERK pathway. So, we speculate whether it could affect the expression of these stemness factors. Western blot showed that FAM289 increased the expression of stemness factors in U251 cell tumor-sphere, including SOX2, OCT4, and c-Myc, whereas FAM289 knockdown dramatically decreased its stemness expression in U87-MG cell tumor-sphere (Fig. 6E). Collectively, this data implies that FAM289-mediated TMZ resistance is through stem-like property acquisition.

### High FAM289 expression in GBM tissues correlated with poor prognosis

**Fig. 7:**
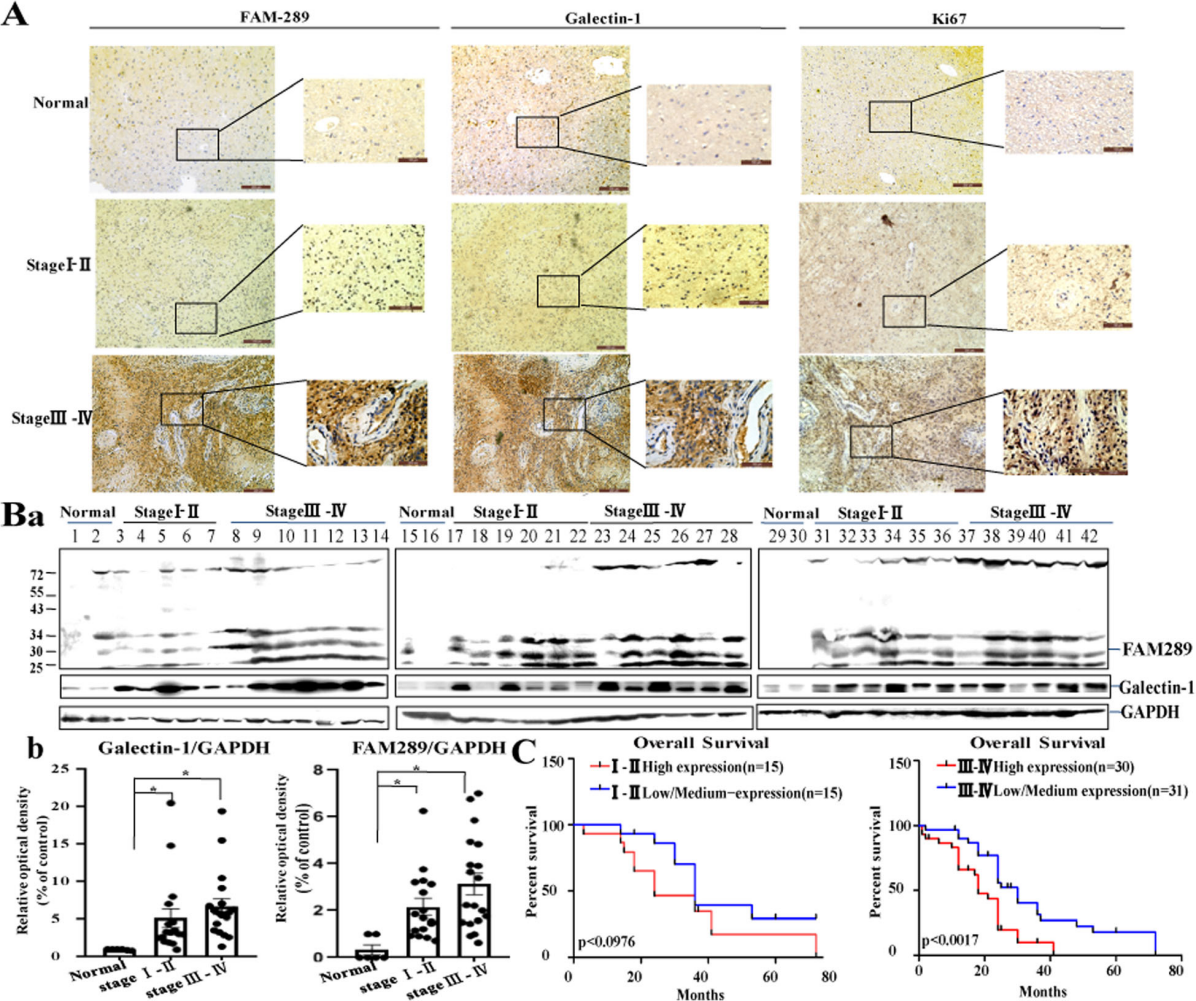
High FAM289 expression in GBM tissues correlated with poor prognosis. (A) Representative images of FAM289, galectin-1 and Ki67 protein expression in GBM tumor tissues and normal brain tissue by immunohistochemistry. (B) FAM289 and Galectin-1 protein in fresh-frozen specimens from normal human brain and GBM tissues was detected by western blotting. GAPDH was used as a loading control. *P < 0.05 vs control. (C) Kaplan–Meier analysis of overall survival curves of in early-stage (I–II) and advanced-stage (III–IV) GBM patients with high FAM289 expression versus low FAM289 expression

Although, the TCGA public datasets imply that high expression of FAM92A1 may be associated with the progression and metastasis of GBM, this is not a direct evidence that FAM289 is one of its transcriptional variants and plays an important role in the prognosis of glioma patients. To further confirm the relationship of FAM289 and GBM, we analyzed FAM289 expression in normal human brain and GBM tissues. As shown in immunohistochemistry (Fig. 7A), FAM289 expression was observed in GBM tissues. In contrast, low FAM289 expression was observed in normal brain tissues (*n* = 6). FAM289 expression in early-stage (I–II) (*n* = 30) and advanced-stage (III–IV) (*n* = 61) GBM tissues was significantly higher than that in normal brain tissues. In addition, FAM289 expression in advanced-stage (III–IV) GBM tissues was significantly higher than that in early-stage (I–II) GBM tissues.

Because FAM92A1 antibody could recognize proteins expressed by other transcriptional variants of FAM92A1 (Including transcriptional variant of FAM289), it is necessary to further confirm the results of immunohistochemistry. Thirty-six patients (early-stage (I–II) (*n* = 19) and advanced-stage (III–IV) (*n* = 17)) with high FAM289 expression were selected from 91 glioma patients by immunohistochemical analysis and confirmed by western blot (Fig. 7Ba). The results show that the expression of FAM289 increased 6.54 times in early-stage (I–II) GBM tissues (*p* < 0.05) and 9.48 times in advanced-stage (III–IV) GBM tissues (*p* < 0.05), and the expression trend of Galectin-1 was similar to that of FAM289 (Fig. 7Bb). Importantly, when early-stage (I–II) and advanced-stage (III–IV) GBM patients were divided into high and low FAM289 expression groups according to the median value of FAM289 levels, the Overall Survival in the high FAM289 expression of advanced-stage (III–IV) GBM group was shorter than that in the low FAM289 expression group (*P* < 0.0017) (Fig. 7Bc). However, there was no significant difference between two groups at early-stage (I–II) GBM patients (*P* < 0.0976). Taken together, these data suggested that the increase of FAM289 in GBM tissues may act as an oncogene and could be served as an independent predictor for overall survival in patients with GBM.

## Discussion

FAM289 (GenBank Accession No. A1XBS5) is a newly-cloned transcriptional variant of FAM92A1, which was clarified in our previous study [9]. FAM92A1, also called Family with Sequence Similarity 92 Member A (FAM92A), belongs to the family of proteins with conserved BAR domain and encodes a group of small-molecule proteins [10, 11]. FAM289 is the largest variant of FAM92A1 encoding a protein of 289 amino acids and containing the complete BAR domain, which has been implicated in tumorigenesis and other cellular processes [16–19]. Alternative splicing is widespread in the human genome and the expression of splicing variants is highly correlated with human diseases, such as cancer [35]. The oncogenic properties of FAM289 have been verified in our previous studies mainly in cervical carcinoma cells [17] and renal tumor cells [18]. In the present study, on the basis of confirming the expression of FAM289 in malignant glioma cells and GBM tissues, the tumorigenic effect of FMA289 was revealed and the related mechanism was explored in FAM289-knocked down and FAM289-overexpressed GBM cells.

The expression of FAM289 in both mRNA and protein levels was positively correlated with different malignant degrees of GBM cells, i.e.U251<DBTRG<U87-MG. FAM289 enhanced cell proliferation and migration in GBM cells. In accordance with in vitro data, FAM289 promoted tumor growth in mouse xenograft models; whilst its knockdown markedly suppressed xenografted tumor growth. In order to explore the mechanisms underlying FAM289-mediated tumor progression, we identified the FAM289 protein interacting with galectin-1 protein. Galectin-1, classified as a proto-type galectin, is well known to be associated with GBM progression via processes of migration [25], invasion [26], angiogenesis [27, 28] and immune escape [29]. It has been reported that protein–protein interaction could affect the tumor-promotion function of galectin-1 [36], implying that the interaction of FAM289 with some proteins may result in the gain of its tumor-promoting properties in GBM. Our results showed that FAM289 and Galectin-1 interdependently regulates tumor proliferation, migration and invasion. Galectin-1 regulates malignant behavior of tumors by the regulation of the MEK/ERK, NF-κB, PI3K/Akt, SAPK/JNK and other important signal pathways [37–39]. Therefore, we next investigated whether FAM289 could modulate these pathways. The results showed that FAM289 regulates proliferation and migration of glioma cells via ERK and NF-κB pathways, which depends on the protein-protein interaction between FAM289 and Galectin-1. NF-κB and ERK are very important protein complexes that control the transcription of their target genes, and they play a critical role in cancer development [37, 38]. Sunahori et al.’s study had found that ERK can induce DNMTS expression [40]. Next, we also found the expression of DNMT1 and DNMT3B were regulated by FAM289. DNMT (DNA methyl transferases), polycomb group genes and histone deacetylases are involved in cell fate determination [41]. DNA methylation, mediated by the combined action of three DNMTs (Dnmt1, Dnmt3a and Dnmt3b), has a particularly prominent role in tumor initiation, progression and specific tumor cell subsets [42]. Studies in glioma had also found that elevated DNMT levels are associated with the tumor suppressor gene hypermethylation and stem cell subsets, linking DNMT activity with tumor-propagating cell populations [43, 44]. However, the expression DNMTs could be regulated by both the ERK and NF-kB pathways [40, 44]. So, our further results showed that FAM289 regulated DNMTs expression depended on ERK activation rather than NF-κB activation, which depends on the interaction between FAM289 protein and Galectin-1 protein.

It has been reported that Galectin-1 can regulate the ERK signaling pathway in many ways, not only determining its expression, but also determining its protein localization in cells. Cytoplasmic Galectin-1 could bind oncogenic H-Ras to mediate Ras membrane anchorage and trigger the Ras/ERK pathway [45–47]. Meanwhile, another study found that nuclear localization of Galectin-1 regulates mammary morphogenesis and promotes breast cancer progression by up-regulation of Erk1/2 expression [22]. In the present study, we found that FAM289 and Galectin-1 did not affect each other’s expression. So, we speculated whether FAM289 regulates malignant behavior of tumors by affecting the intracellular localization of Galectin-1. However, we were surprised to find that Galectin-1 could affect the intracellular localization of FAM289 protein and facilitate its entry into the nucleus, rather than FAM289 affecting the intracellular localization of Galectin-1protein. Nuclear FAM289-Galectin-1 interaction controls the activation of the ERK pathway to upregulate DNMTs expression and the tumor-promotion effects of FAM289 in malignant glioma.

Studies have shown that Galectin-1 knockdown decreases TMZ resistance in glioblastoma [48–50]. Our above studies have shown that FAM289 and Galectin-1 proteins are interdependent in regulating malignant behavior of glioma, so we speculated that FAM289 may also have the function to affect TMZ resistance in GBM. The results confirm our hypothesis that FAM289 may affect TMZ resistance by regulating the apoptotic rate and cell cycle of glioma cells. Accumulating evidence has shown that chemotherapy failure might be caused by the existence of cancer stem cells (CSCs) [6, 7, 51]. Our results shown that FAM289 could increase DNMTS expression by activating ERK, Many reports have also suggested that the MEK/ERK pathway could regulate the expression of stemness factors such as SOX2, OCT4 and c-Myc by inducing DNMTs expression [33, 34, 52–55]. As we expected, we found that FAM289-mediated TMZ resistance is through stem-like property acquisition by activating ERK, which may be the key reason for its oncogenic role in GBM.

More importantly, the clinical implication of FAM289 was assessed in the normal and glioma patients’ brain tissue, which demonstrated that high expression of FAM289 predicts adverse prognosis in advanced-stage GBM patients and cells, consistent with its putative pro-tumorigenic role in GBM. Herein, the expression of FAM289 was positively correlated with Galectn-1, suggesting that FAM289 can be used in association with Galectin-1 as a complementary diagnostic tool to improve discrimination between benign and malignant gliomas. Our observations validate and further support the potential value of FAM289 in the diagnosis of well differentiated GBM cancer.

## Conclusion

**Fig. 8:**
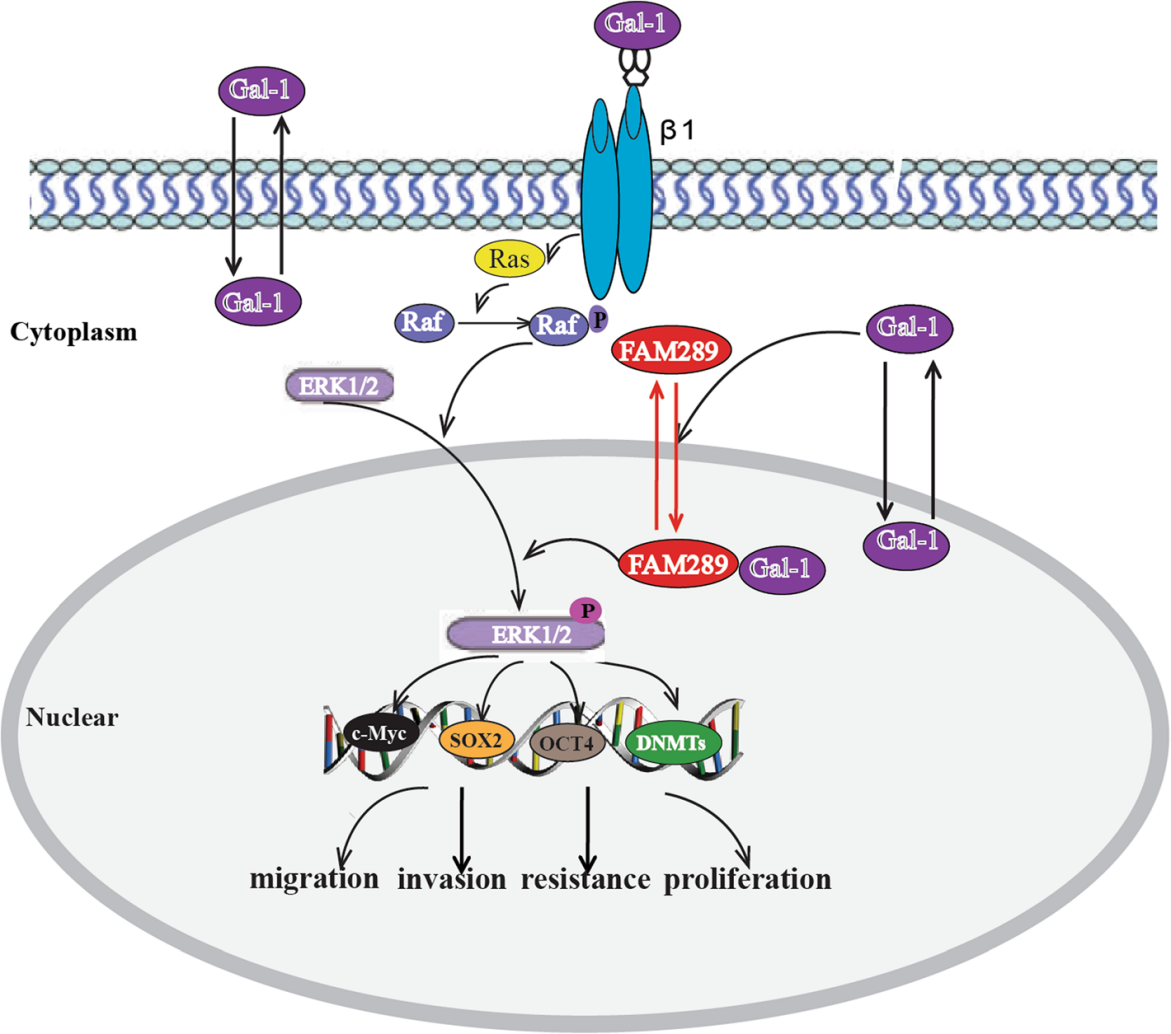
Schematic representation of the proposed mechanism of FAM289 in GBM cells. FAM289 contributes to tumorigenesis in malignant glioma by interacting with Galectin-1 to promote FAM289 protein translocation into cell nucleus, which could activate the ERK pathway to up regulate DNMTs expression and increase stem-like property gene expression, thereby affecting its drug sensitivity of TMZ in the treatment of glioma. Gal-1: Galectin-1, β1: β1 integrin, p: phosphorylation

In summary, FAM289 is a novel oncogene involved in GBM through increasing cell proliferation, inhibiting apoptosis and promoting stem-like properties. We demonstrated for the first time that FAM289 contributes to tumorigenesis in malignant gliomas by interacting with Galectin-1 to promote FAM289 protein into the cell nucleus. The protein-protein interaction between FAM289 and Galectin-1 could activate the ERK pathway to up regulate DNMTs expression and increase stem-like properties gene expression, and thus affecting its drug sensitivity of TMZ in the treatment of gliomas (Fig. 8). This study provided functional evidence for FAM289 to be developed as a therapeutic target for cancer treatment.

## Additional files

Additional file 1: **Figure S1.** Analysis of Ki67 expression to identify the construction of glioma orthotopic model in NCG mice by immunohistochemistry. The arrow shows the Ki67 positive region. (TIF 524 kb)

Additional file 2: **Figure S2.** Knockdown or overexpression of Galectin-1 in U251 or U87-MG cells. A. Galectin-1 was knocked down in U251 cell lines by SiRNA1–3. Galectin-1 siRNA2 and siRNA3 significantly reduced Galectin-1expression. B. Overexpression of Galectin-1-Flag fusion protein in U87-MG cells was verified by western blotting. (TIF 118 kb)

Additional file 3: **Figure S3.** Screening of promoting tumor proliferation, migration or downstream of ERK and NF-kB genes expression regulated by FAM289 on by RT-PCR. (TIF 528 kb)

Additional file 4: **Figure S4.** The effect of FAM289 and Galectin-1 on each other’s expression. A. Overexpression FAM289 or Galectin-1 did not affect each other’s expression. B. Knock down FAM289 or Galectin-1 did not affect each other’s expression. (TIF 69 kb)

Additional file 5: **Figure S5.** Localization of FAM289 constructs. Fluorescence micrographs showed the subcellular localization of each fusion protein pEGFP-FAM289, pEGFP-FAM289-NLS and pEGFP-FAM289-NES (green) in U251 cells, Scale bar, 50 μm. (TIF 315 kb)

Additional file 6: The list of primers and antibodies. (DOCX 33 kb)

## Data Availability

All data generated or analyzed during this study are included in this published article (and its supplementary information files).

## Abbreviations

Co-IP: Co-immunoprecipitation
Con: Control
GBM: Glioblastoma multiforme cells
IHC: Immunohistochemistry
IVIS: In vivoimaging systems
KD: Knock down
NABT: Normal brain tissue
OE: Overexpression
qRT-PCR: Quantitative real-time PCR
siRNA: Short interfering RNA
TMZ: Temozolomide
