# Supplementary figures and images for: Nuclear FAM289-Galectin-1 interaction controls FAM289-mediated tumor promotion in malignant glioma
Source: J Exp Clin Cancer Res. 2019 Sep 6;38:394. doi: 10.1186/s13046-019-1393-7 (PMC6731628; doi:10.1186/s13046-019-1393-7)

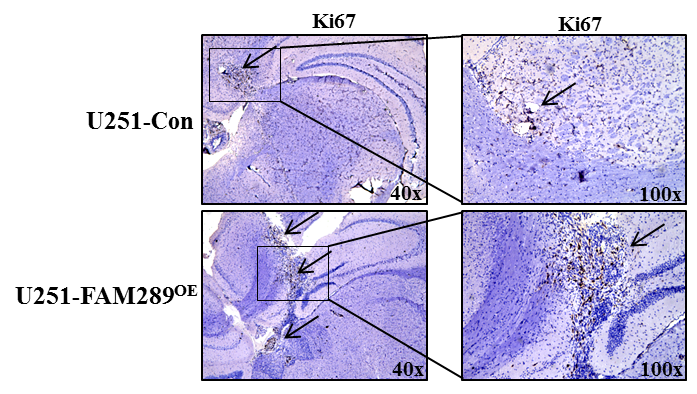

Supplement: Supplementary file 1 — Figure S1. Analysis of Ki67 expression to identify the construction of glioma orthotopic model in NCG mice by immunohistochemistry. The arrow shows the Ki67 positive region. (TIF 524 kb) [file 13046_2019_1393_MOESM1_ESM.tif]

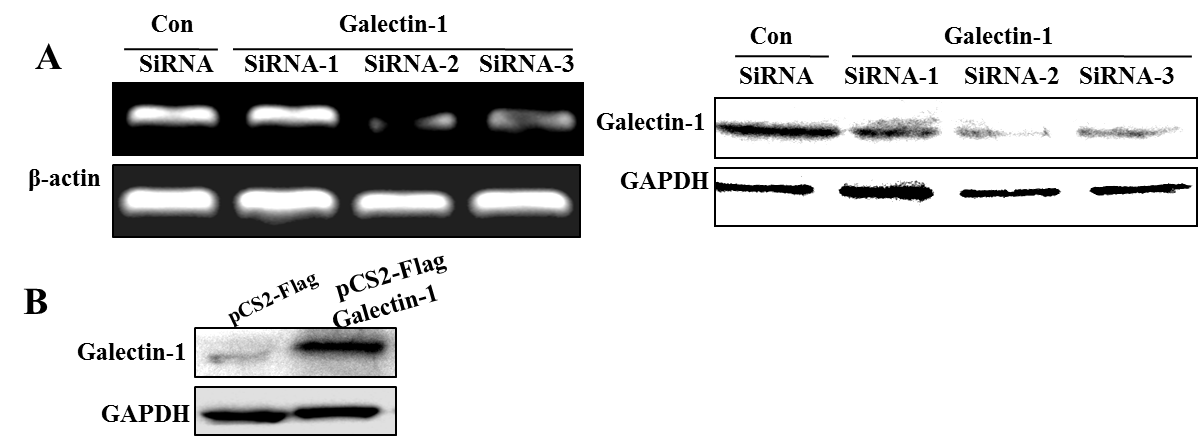

Supplement: Supplementary file 2 — Figure S2. Knockdown or overexpression of Galectin-1 in U251 or U87-MG cells. A. Galectin-1 was knocked down in U251 cell lines by SiRNA1–3. Galectin-1 siRNA2 and siRNA3 significantly reduced Galectin-1expression. B. Overexpression of Galectin-1-Flag fusion protein in U87-MG cells was verified by western blotting. (TIF 118 kb) [file 13046_2019_1393_MOESM2_ESM.tif]

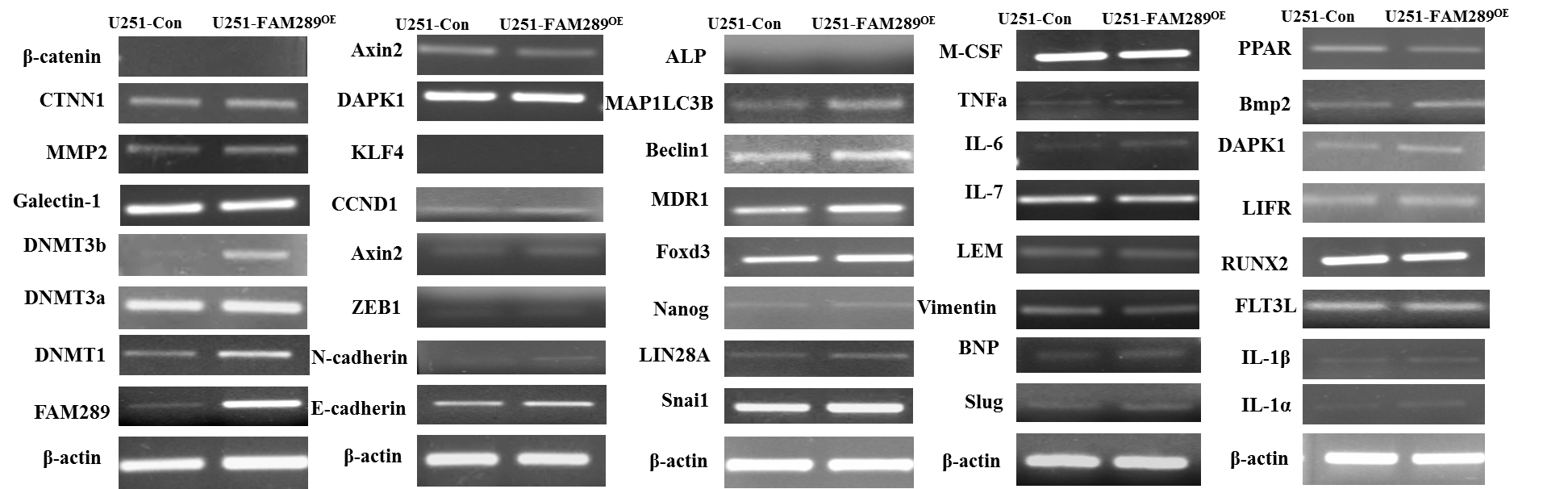

Supplement: Supplementary file 3 — Figure S3. Screening of promoting tumor proliferation, migration or downstream of ERK and NF-kB genes expression regulated by FAM289 on by RT-PCR. (TIF 528 kb) [file 13046_2019_1393_MOESM3_ESM.tif]

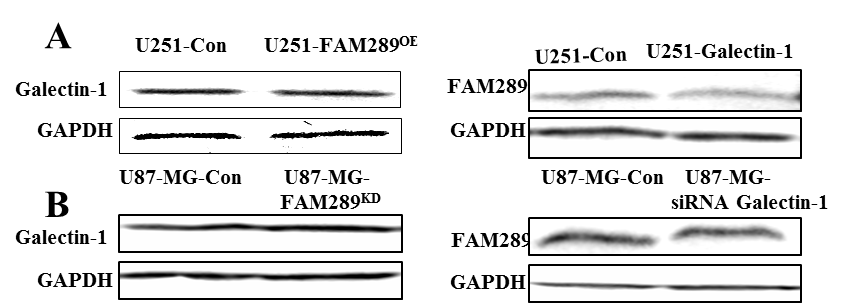

Supplement: Supplementary file 4 — Figure S4. The effect of FAM289 and Galectin-1 on each other’s expression. A. Overexpression FAM289 or Galectin-1 did not affect each other’s expression. B. Knock down FAM289 or Galectin-1 did not affect each other’s expression. (TIF 69 kb) [file 13046_2019_1393_MOESM4_ESM.tif]

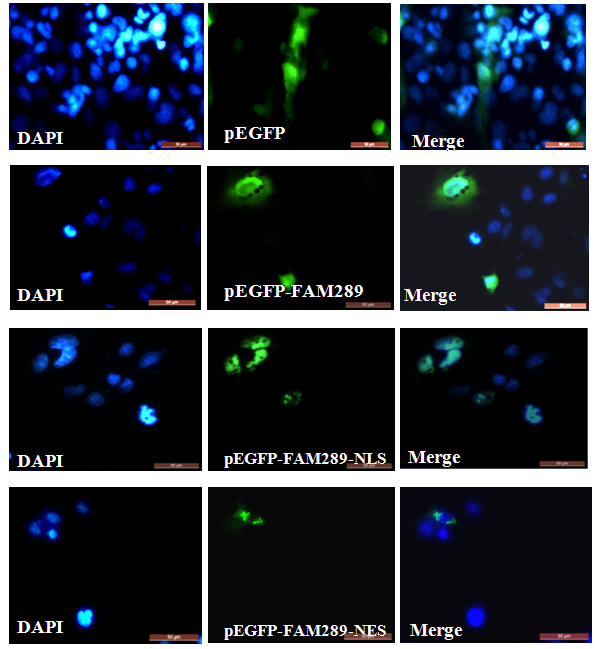

Supplement: Supplementary file 5 — Figure S5. Localization of FAM289 constructs. Fluorescence micrographs showed the subcellular localization of each fusion protein pEGFP-FAM289, pEGFP-FAM289-NLS and pEGFP-FAM289-NES (green) in U251 cells, Scale bar, 50 μm. (TIF 315 kb) [file 13046_2019_1393_MOESM5_ESM.tif]
