# Supplementary material for: Nuclear FAM289-Galectin-1 interaction controls FAM289-mediated tumor promotion in malignant glioma
Source: J Exp Clin Cancer Res. 2019 Sep 6;38:394. doi: 10.1186/s13046-019-1393-7 (PMC6731628; doi:10.1186/s13046-019-1393-7)
Supplement: Supplementary file 6 — The list of primers and antibodies. (DOCX 33 kb) [file 13046_2019_1393_MOESM6_ESM.docx]

|  | |
| --- | --- |
| Additional file 6  **Table S1.** primer sequences for FAM289 lenti-CRISPRv2/Cas9 plasmid constructs   \| **Primer names** \| **Sequences** \| \| --- \| --- \| \| sgRNA-1 forward \| 5'-CACCGACGGGGATTCCAAGGCGCCT-3' \| \| sgRNA-1 reverse \| 5'-AAACAGGCGCCTTGGAATCCCCGTC-3' \| \| sgRNA-2 forward \| 5'-CACCGAAGGCGCCTGGGCGCGCGCT-3' \| \| sgRNA-2 reverse \| 5'-AAACAGCGCGCGCCCAGGCGCCTTC-3' \| \| sgRNA-3 forward \| 5'-CACCGACGCGTTAATTTCATTCACC-3' \| \| sgRNA-3 reverse \| 5'-AAACGGTGAATGAAATTAACGCGTC-3' \|   **Table S2.** siRNA target sequences for RNA interference   \| Target gene names \| siRNA target sequences \| \| --- \| --- \| \| FAM289 siRNA-1 \| 5′-GTATCCACTTGTCGACTAA-3′ \| \| FAM289 siRNA-2 \| 5′-GCAGAAACGGAATTACAGA-3′ \| \| FAM289 siRNA-3 \| 5′-TGATGAGGATGACGAGTTA-3′ \| \| Galectin-1 siRNA-1 \| 5′-GCTGCCAGATGGATACGAA-3′ \| \| Galectin-1 siRNA-2 \| 5′-CCAACACCATCGTGTGCAA-3′ \| \| Galectin-1 siRNA-3 \| 5′-GACGGTGACTTCAAGATCA-3′ \| \| siRNA Con (GFP) \| 5′-CTTACGCTGAGTACTTCGA-3′ \|   **Table S3. Sequences of primers used for q-RTPCR** | |
| **Primer names** | **Sequences** |
| **Sequences of primers used for RT-qPCR** |  |
| 1 β-actin forward | 5'-CCTCGCCTTTGCCGATCC-3' |
| 1 β-actin reverse | 5'-GGATCTTCATGAGGTAGTCAGTC-3' |
| 2 DNMT1 forward | 5'- TACCTGGACGACCCTGACCTC-3' |
| 2 DNMT1 reverse | 5'- CGTTGGCATCAAAGATGGACA-3' |
| 3 DNMT3a forward | 5'-TATTGATGAGCGCACAAGAGAGC |
| 3 DNMT3a reverse | 5'-GGGTGTTCCAGGGTAACATTGAG |
| 4 DNMT3b forward | 5'-GGCAAGTTCTCCGAGGTCTCTG-3'' |
| 4 DNMT3b reverse | 5'- TGGTACATGGCTTTTCGATAGGA-3' |
| 5 FAM289 forward | 5’- GAACGCTCAAACGAAACAA-3’ |
| 5 FAM289 reverse | 5’- TCACAATGGTCCCATAAGT-3’ |
| 6 Galectin-1 forward | 5'-cttgtggtctggtcgccagc-3' |
| 6 Galectin-1 reverse | 5'-agcgagggttgaagtgcagg-3' |
| 7 MMP2 forward | 5'-CACTTTCCTGGGCAACAAAT-3' |
| 7 MMP2 reverse | 5'-TGATGTCATCCTGGGACAGA-3' |
| 8 CTNN1 forward | 5'-AAAGCGGCTGTTAGTCACTGG-3' |
| 8 CTNN1 reverse | 5'-CGAGTCATTGCATACTGTCCAT-3' |
| 9 β-catenin forward | 5'-TTCACTCTAGGAATGAAGGTGTGG-3' |
| 9 β-catenin reverse | 5'-CGTTTCTTGTAATCTTGTGGCTTG-3' |
| 10 E-cadherin forward | 5'-CGAGAGCTACACGTTCACGG-3' |
| 10 E-cadherin reverse | 5'-GGGTGTCGAGGGAAAAATAGG-3' |
| 11 N-cadherin forward | 5'-TTTGATGGAGGTCTCCTAACACC-3' |
| 11 N-cadherin reverse | 5'-ACGTTTAACACGTTGGAAATGTG-3' |
| 12 ZEB1 forward | 5'-GATGATGAATGCGAGTCAGATGC-3' |
| 12 ZEB1 reverse | 5'-ACAGCAGTGTCTTGTTGTTGT-3' |
| 13 Axin2 forward | 5'-CAACACCAGGCGGAACGAA-3' |
| 13 Axin2 reverse | 5'-GCCCAATAAGGAGTGTAAGGACT-3' |
| 14 CCND1 forward | 5'-GCTGCGAAGTGGAAACCATC-3' |
| 14 CCND1 reverse | 5'-CCTCCTTCTGCACACATTTGAA-3' |
| 15 KLF4 forward | 5'-CAGCTTCACCTATCCGATCCG-3' |
| 15 KLF4 reverse | 5'-GACTCCCTGCCATAGAGGAGG-3' |
| 16 DAPK1 forward | 5'-GAGTTTGTCGCTCCTGAGATAGT-3' |
| 16 DAPK1 reverse | 5'-GCTTAGTGTCTCCAAGAAATGGG-3' |
| 17 Axin2 forward | 5'-CAACACCAGGCGGAACGAA-3' |
| 17 Axin2 reverse | 5'-GCCCAATAAGGAGTGTAAGGACT-3' |
| 18 Snai1 forward | 5'-TCGGAAGCCTAACTACAGCGA-3' |
| 18 Snai1 reverse | 5'-AGATGAGCATTGGCAGCGAG-3' |
| 19 LIN28A forward | 5'-TGCGGGCATCTGTAAGTGG-3' |
| 19 LIN28A reverse | 5'-GGAACCCTTCCATGTGCAG-3' |
| 20 Nanog forward | 5'-TTTGTGGGCCTGAAGAAAACT-3' |
| 20 Nanog reverse | 5'-AGGGCTGTCCTGAATAAGCAG-3' |
| 21 Foxd3 forward | 5'-AGGGAAGTTTGGTCAATCAGAA-3' |
| 21 Foxd3 reverse | 5'-TGGAGATGAGGGAATCAAAGTT-3' |
| 22 MDR1 forward | 5'-CCCATCATTGCAATAGCAGG-3' |
| 22 MDR1 reverse | 5'-GTTCAAACTTCTGCTCCTGA-3' |
| 23 Beclin1 forward | 5'-AGGTTGTAGAAAGGCGAGACA-3' |
| 23 Beclin1 reverse | 5'-GTGGTTTCAAGGCCAGATGT-3' |
| 24 MAP1LC3B forward | 5'-CTCGAGATGCCGTCGGAG-3' |
| 24 MAP1LC3B reverse | 5'-GAATTCGCACTGACAATTTC-3' |
| 25 ALP forward | 5'-GACGGTGAACGGGAGAAC-3' |
| 25 ALP reverse | 5'-CTCAGAACAGGGTGCGTAG-3' |
| 26 slug forward | 5'-ATCTGACCCGTCGTGACG-3' |
| 26 slug reverse | 5'-CGTCACGACGGGTCAGAT-3' |
| 27 BNP forward | 5'-ATGGATCCAGCCCCAAGATGGTG-3' |
| 27 BNP reverse | 5'-AAGACGTCTTAACAATGCCGCCTCAGC-3' |
| 28 Vimentin forward | 5'-ACCATTAACAGGAACACAGG-3' |
| 28 Vimentin reverse | 5'-CAGTCACTTTCAGTGTGGTG-3' |
| 29 LEM forward | 5'-AAGGGCAAGCCAAGTCTTTGA-3' |
| 29 LEM reverse | 5'-CGGCTAATCAGGTCCTCTATCTG-3' |
| 30 IL-7 forward | 5'-TCGTCCACGGGTCCTCTCTCCC-3' |
| 30 IL-7 reverse | 5'-GTACAGTTTTGTAAACGATGG-3' |
| 31 IL-6 forward | 5'-CCTTCGGTCCAGTTGCCTTCT-3' |
| 31 IL-6 reverse | 5'-CCAGTGCCTCTTTGCTGCTTTC-3' |
| 32 TNFa forward | 5'-GAGGCCAAGCCCTGGTATG-3' |
| 32 TNFa reverse | 5'-CGGGCCGATTGATCTCAGC-3' |
| 33 M-CSF forward | 5'-CTTGCCATGGAGGAGGTGTCGGAGTACTG-3' |
| 33 M-CSF reverse | 5'-ATGTGTCGACCTCCTGGAGCTGCGGGCTG-3' |
| 34 IL-1α forward | 5'-GGAATTCATGGCCAAAGTTCCA-3' |
| 34 IL-1α reverse | 5'-GGTCGACCTACGCCTGGTTTC-3' |
| 35 IL-1β forward | 5'-CTCCGGGACTCACAGCAAA-3' |
| 35 IL-1β reverse | 5'-GGGAACTGGGCAGACTCAAA-3' |
| 36 FLT3L forward | 5'-TGGAGCCCAACAACCTATCTC-3' |
| 36 FLT3L reverse | 5'-ACGGATTTTGACAGCGAAGTC-3' |
| 37 RUNX2 forward | 5'-TGGTTACTGTCATGGCGGGTA-3' |
| 37 RUNX2 reverse | 5'-TCTCAGATCGTTGAACCTTGCTA-3' |
| 38 LIFR forward | 5'-GTGACCCACAACACAACTCTG-3' |
| 38 LIFR reverse | 5'-CACATTCCAAGGGCATATCTGAG-3' |
| 39 DAPK1 forward | 5'-GAGTTTGTCGCTCCTGAGATAGT-3' |
| 39 DAPK1 reverse | 5'-GCTTAGTGTCTCCAAGAAATGGG-3' |
| 40 Bmp2 forward | 5'-GGCACTGGGATGTCTACTCTA-3' |
| 40 Bmp2 reverse | 5'-CCATCACAGATAGCAACCTGACT-3' |
| 41 PPAR forward | 5'-GATGCCAGCGACTTTGACTC-3' |
| 41 PPAR reverse | 5'-ACCCACGTCATCTTCAGGGA-3' |
| 42 Nrf2 forward | 5'-TCAGCGACGGAAAGAGTATGA-3' |
| 42 Nrf2 reverse | 5'-CCACTGGTTTCTGACTGGATGT-3' |

**Table S4. Antibodies used for western blotting (WB), Co-immunoprecipitation assay (Co-IP) and flow cytometry (FC), Immunohistochemistry (IHC), Immunofluorescence (IF)**

| **Protein** | **Applications** | **Antibody** | **Origin** | **dilution** | **Molecular weight** |
| --- | --- | --- | --- | --- | --- |
| GAPDH | WB | Proteintech, USA | Mous | 1:1000 | 36 KDa |
| GFP | WB | Sigma, USA | Mous | 1:1000 | 27KDa |
| FALG | WB | Sigma, USA | Rabbit | 1:1000 | 2.7 KDa |
| FAM289(FAM92A1) | WB IHC | Atlas, USA | Rabbit | 1:1000 | 30KDa |
| FAM289(FAM92A1) | IF, IP | Proteintech, USA | Rabbit | 1:200, 1:100 | 30KDa |
| Galectin-1 | WB, IF | Proteintech, USA | Rabbit | 1:1000, 1:200 | 15KDa |
| H3 | WB | Proteintech, USA | Rabbit | 1:1000 | 15KDa |
| ERK1/2 | WB | Proteintech, USA | Rabbit | 1:1000 | 44/42KDa |
| NF-κB | WB | Proteintech, USA | Rabbit | 1:1000 | 60KDa |
| DNMT1 | WB | Proteintech, USA | Rabbit | 1:1000 | 180KDa |
| DNMT3B | WB | Proteintech, USA | Rabbit | 1:1000 | 95KDa |
| SOX2 | WB | Affinity,China | Mouse | 1:500 | 35KDa |
| Oct4 | WB | Proteintech, USA | Mouse | 1:100 | 45KDa |
| CD133 | WB | Proteintech, USA | Mouse | 1:500 | 97KDa |
| c-Myc | WB | Cell Signaling Technology | Rabbit | 1:1000 | 62KDa |
| pERK1/2 | WB | Cell Signaling Technology | Rabbit | 1:1000 | 44/42KDa |
| pNF-κB | WB | Cell Signaling Technology | Rabbit | 1:1000 | 60 KDa |
| IgG | Co-IP | ab18413, Abcam | Mouse | 1:10 | 150 KDa |
| Ki67-PE | FC | Biolegend, USA | Mouse | 5ul/test |  |
| CXCR4-PE | FC | Biolegend, USA | Mouse | 5ul/t est |  |
| CD133-PE | FC | Biolegend, USA | Mouse | 5ul/test |  |

**Table S5 Sequences of primers used for plasmid construction**

| pCS2-3Flag-FAM289 forward | 5'-ccgggccggccCATGATGAGGCGCACCCTGGA-3' |
| --- | --- |
| pCS2-3Flag-FAM289 reverse | 5'-ttggcgcgccTTACTTAAGAAAATTTTCTT-3' |
| NLS-GEMIN4-FAM289 forward | 5'-CCGGAATTCatgccccatcctccaaagaggcttaggtcagacccagacgcgtgcATGATGAGGCGCACC-3' |
| NES-STAU2-FAM289 forward | 5'-CCGGAATTCATGataaatcagatgttctcagtgcagctgagtcttATGATGAGGCGCACC-3' |
| FAM289-eGFP reverse | 5'-CCGCTCGAGttacttgtacagctcgtccatgccgagagtgatccc-3' |
| pCS2--3FlagGalectin forward | 5'-ATGCGGCCGGCCGATGGCTTGTGGTCTGGT-3' |
| pCS2--3Flag Galectin reverse | 5'-ATGCGGCGCGCCTCAGTCAAAGGCCACAC-3' |
| RFP-Galectin-1 forward | 5'-CCGGAATTCATGGCTTGTGGTCTGGT-3' |
| RFP-Galectin-1reverse | 5'-CCGCTCGAGTCAGTCAAAGGCCACAC-3' |
